# Combined deletion of Pten and p53 in mammary epithelium accelerates triple-negative breast cancer with dependency on eEF2K

**DOI:** 10.15252/emmm.201404402

**Published:** 2014-10-20

**Authors:** Jeff C Liu, Veronique Voisin, Sharon Wang, Dong-Yu Wang, Robert A Jones, Alessandro Datti, David Uehling, Rima Al-awar, Sean E Egan, Gary D Bader, Ming Tsao, Tak W Mak, Eldad Zacksenhaus

**Affiliations:** 1 Division of Advanced Diagnostics, Toronto General Research Institute – University Health NetworkToronto, ON, Canada; 2 The Donnelly Centre, University of TorontoToronto, ON, Canada; 3 Department of Laboratory Medicine & Pathobiology, University of TorontoToronto, ON, Canada; 4 Princess Margaret Cancer CenterToronto, ON, Canada; 5 Campbell Family Institute for Breast Cancer Research, Princess Margaret HospitalToronto, ON, Canada; 6 SMART Laboratory for High-Throughput Screening Programs, Lunenfeld-Tanenbaum Research Institute at Mount Sinai HospitalToronto, ON, Canada; 7 Department of Agricultural, Food and Environmental Sciences, University of PerugiaPerugia, Italy; 8 Drug Discovery Program, Department of Pharmacology and Toxicology, Ontario Institute for Cancer Research, University of TorontoToronto, ON, Canada; 9 Program in Developmental and Stem Cell Biology, The Hospital for Sick ChildrenToronto, ON, Canada; 10 Department of Molecular Genetics, University of TorontoToronto, ON, Canada; 11 Department of Medical Biophysics, University Health NetworkToronto, ON, Canada

**Keywords:** eEF2K, p53, prognosis, Pten, triple-negative breast cancer

## Abstract

The tumor suppressors Pten and p53 are frequently lost in breast cancer, yet the consequences of their combined inactivation are poorly understood. Here, we show that mammary-specific deletion of Pten via WAP-Cre, which targets alveolar progenitors, induced tumors with shortened latency compared to those induced by MMTV-Cre, which targets basal/luminal progenitors. Combined Pten-p53 mutations accelerated formation of claudin-low, triple-negative-like breast cancer (TNBC) that exhibited hyper-activated AKT signaling and more mesenchymal features relative to Pten or p53 single-mutant tumors. Twenty-four genes that were significantly and differentially expressed between WAP-Cre:Pten/p53 and MMTV-Cre:Pten/p53 tumors predicted poor survival for claudin-low patients. Kinome screens identified eukaryotic elongation factor-2 kinase (eEF2K) inhibitors as more potent than PI3K/AKT/mTOR inhibitors on both mouse and human Pten/p53-deficient TNBC cells. Sensitivity to eEF2K inhibition correlated with AKT pathway activity. eEF2K monotherapy suppressed growth of Pten/p53-deficient TNBC xenografts *in vivo* and cooperated with doxorubicin to efficiently kill tumor cells *in vitro*. Our results identify a prognostic signature for claudin-low patients and provide a rationale for using eEF2K inhibitors for treatment of TNBC with elevated AKT signaling.

See also: **HG Russnes & C Caldas *et al*** (December 2014)

## Introduction

Breast cancer (BC) is a heterogeneous disease that can be classified into estrogen receptor α-positive (ERα^+^) and HER2^+^ tumors as well as triple-negative (TN) tumors, which do not express high levels of these or the progesterone receptors (Prat & Perou, 2011). TNBCs include two major subtypes: basal-like, expressing basal-cell markers such as cytokeratin 14, and claudin-low/mesenchymal-like, expressing low levels of tight junction proteins including certain claudins and E-cadherin, and high levels of genes associated with epithelial-to-mesenchymal transition (EMT) (Prat *et al*, 2010; Lehmann *et al*, 2011; Timmerman *et al*, 2013). Interest in the latter tumors is driven by observations that following conventional therapy, residual tumors exhibit features of cancer stem cells and EMT (Mani *et al*, 2008; Creighton *et al*, 2009; Guo *et al*, 2012). Moreover, TNBCs often resist therapy, and metastatic disease is virtually incurable (Carey *et al*, 2007; Irshad *et al*, 2011). While specific treatments have been developed for ERα^+^ BC (tamoxifen, aromatase inhibitors) and HER2^+^ BC (trastuzumab), the only option for most TNBC patients is cytotoxic chemotherapy such as anthracyclines (doxorubicin), which leads to significant morbidity.

In TNBC, p53 is deleted or mutated in 60–80% of cases (Holstege *et al*, 2010; Koboldt *et al*, 2012; Shah *et al*, 2012), whereas the Phosphatase and TENsin (Pten) homolog deleted in chromosome 10 (Li *et al*, 1997; Steck *et al*, 1997) is lost in 25–30% of cases primarily through promoter silencing or microRNA-mediated suppression (Salmena *et al*, 2008; Korkaya *et al*, 2009; Koboldt *et al*, 2012). The protein, Pten, regulates cell growth by converting phosphatidylinositol (3,4,5)-trisphosphate (PIP3) into phosphatidylinositol (4,5)-disphosphate (PIP2), thereby antagonizing phosphatidylinositol-3 kinase (PI3K) pathway activation (Stambolic *et al*, 1998; Cully *et al*, 2006; Adams *et al*, 2011). Dysregulation of the PI3K pathway induces AKT/PKB, leading to increased cell motility, proliferation and survival, as well as increased protein translation via mTOR. Pten and p53 were shown to regulate EMT and cell migration (Leslie *et al*, 2007; Jiang *et al*, 2011), and interact with each other at several levels (Stambolic *et al*, 2001; Kawase *et al*, 2009).

While p53 loss is not actionable, activation of the PI3K pathway can be targeted with PI3K pathway antagonists such as PI3K, AKT or mTOR inhibitors (Janku *et al*, 2012; Kim *et al*, 2012). However, as the PI3K pathway is subject to tight autoregulation, such inhibitors often have modest or transient effects (Gordon & Banerji, 2013). There is therefore an urgent need to identify new therapeutic targets that may be useful for treatment of Pten/p53-deficient TNBC. The effects of mutations in p53 or Pten on the mammary epithelium have been documented (Stambolic *et al*, 2000; Li *et al*, 2002; Herschkowitz *et al*, 2012; Knight *et al*, 2013). The impact of combined inactivation of these tumor suppressors, which frequently occurs in breast cancer, is poorly understood. Here, we disrupted Pten and p53 in mammary epithelium either alone or in combination and determined the effect on tumor formation, tumor-initiating cells, prognosis, PI3K/AKT pathway activation and response to therapeutic drugs. We found that Pten/p53 deficiency induces TNBCs, which are distinct from Pten or p53 single-mutant tumors with more mesenchymal features and poor clinical outcome. A non-biased screen revealed that while PI3K/AKT/mTOR inhibitors efficiently kill Pten/p53-deficient tumors, the most potent drugs target JNK, which was previously linked to Pten-deficient cancer, and eEF2K, a kinase that controls protein translation downstream of mTOR. Sensitivity to eEF2K was proportional to AKT pathway activity and was demonstrated both *in vitro* and in xenografts of mouse and human Pten/p53-deficient TNBC. Our results should encourage development of effective eEF2K inhibitors for treatment of TNBC with elevated AKT signaling.

## Results

### Combined deletion of Pten and p53 induces spindle-/mesenchymal-like mammary tumors

**Figure 1:**
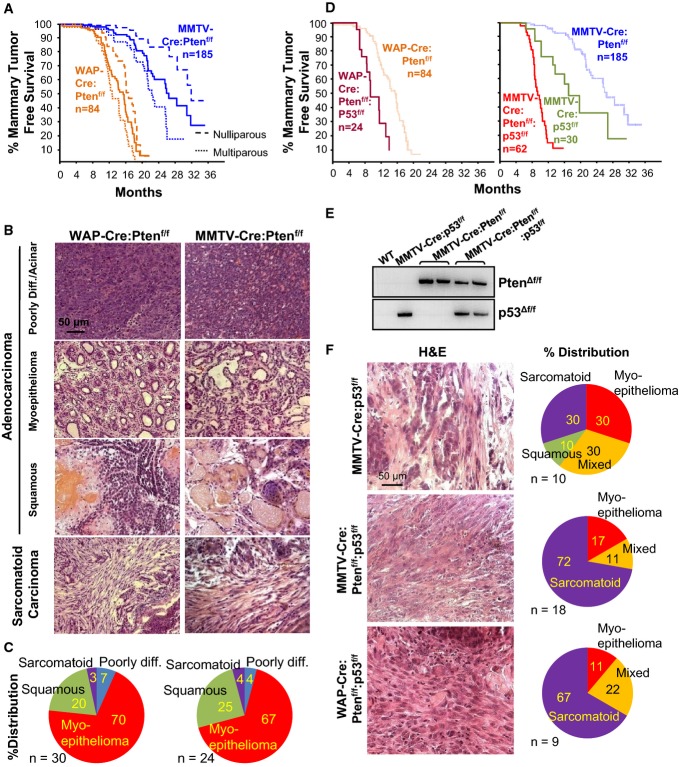
Pten plus p53 mutations cooperate to accelerate sarcomatoid/mesenchymal-like mammary tumors Kaplan–Meier mammary tumor-free curves for WAP-Cre:Pten^f/f^ and MMTV-Cre:Pten^f/f^ mice. Dashed lines represent nulliparous or multiparous females and solid line the average for all mice. Tumor latency (average) for the two models was significantly different (*P* = 3.47 × 10^−17^, Wilcoxon method).Histology of four major tumor types in WAP-Cre:Pten^f/f^ and MMTV-Cre:Pten^f/f^ mice.Distribution of tumor types (%) in WAP-Cre:Pten^f/f^ (left) and MMTV-Cre:Pten^f/f^ (right) mice.Kaplan–Meier mammary tumor-free curves for WAP-Cre:Pten^f/f^:p53^f/f^, MMTV-Cre:p53^f/f^ and MMTV-Cre:p53^f/f^ versus (average) WAP-Cre:Pten^f/f^ and MMTV-Cre:Pten^f/f^ mice. Statistical significance by Wilcoxon method. p53 versus Pten, *P* = 0.00158; Pten/p53 versus p53, *P* = 0.0329; Pten/p53 versus Pten, *P* = 4.71 × 10^−14^.Detection of Pten and p53 gene deletion by PCR using primers specific for Cre-excised Pten^f/f^ and p53^f/f^ alleles.Histology of indicated tumors and distribution (%) of tumor types.

To model the effect of Pten loss on BC, we used a floxed allele (Pten^f^) (Suzuki *et al*, 1998) and the deleter lines WAP-Cre (which preferentially targets pregnancy-identified alveolar progenitors) and MMTV-Cre^NLST^ (which targets basal and luminal progenitors) (Wagner *et al*, 2002; Jiang *et al*, 2010). MMTV-Cre:Pten^f/f^ mice developed mammary tumors after a long latency of 26.4 months with incomplete penetrance (70%) (Fig1A). WAP-Cre:Pten^f/f^ females developed tumors with shorter latency (15.2 months) and almost complete penetrance; by 18 months, nearly all mice had succumbed to cancer. In both cases, pregnancy accelerated tumor formation. Tumors from both models were heterogeneous, consisting primarily of adenomyoepithelioma (∼70%) or adenosquamous carcinoma (20–25%) (Fig1B and C). In addition, a small fraction of tumors was classified as acinar or poorly differentiated adenocarcinoma (4–7%), or spindle-cell/adenosarcoma (3–4%). Marker expression analysis of the dominant tumor subtypes revealed mixed expression of smooth muscle actin (SMA) and cytokeratin 5 (K5), K6, K14 (basal markers), K18 (luminal marker), vimentin, ERα, as well as nuclear co-localization of β-catenin and cyclin D2 (Supplementary Fig S1), a pattern often found in other tumor models of mixed lineages such as MMTV-WNT1 (Li *et al*, 2003).

Next, we determined the effect of concurrent loss of Pten and p53, which are frequently inactivated in TNBC. MMTV-Cre:Pten^f/f^:p53^f/f^ and WAP-Cre:Pten^f/f^:p53^f/f^ double-mutant females developed tumors with a reduced latency of 11.3 and 9.8 months, respectively, compared with 26.4, 15.2 and 16.9 months for single-mutant MMTV-Cre:Pten^f/f^, WAP-Cre:Pten^f/f^ and MMTV-Cre:p53^f/f^ mice (Fig1D). Deletion of the Pten^f/f^ and p53^f/f^ alleles in these tumors was confirmed by PCR (Fig1E). In contrast to the heterogeneity of Pten^Δf^ tumors and small percentage of adenosarcomas, approximately 70% of Pten^Δf^:p53^Δf^ lesions were histologically classified as adeno-sacrcomatoid/spindle-cell/mesenchymal-like BC. The rest exhibited mixed mesenchymal plus adenocarcinomas or differentiated adenocarcinomas (Fig1F). In comparison, only 30% of p53^Δf^ tumors were sarcomatoid. The Pten/p53-deficient adeno-sacrcomatoid-like tumors expressed the mesenchymal markers vimentin, SMA and desmin but not ERα (Supplementary Fig S2).

### Pten/p53-deficient mouse tumors cluster with human claudin-low TNBC

**Figure 2:**
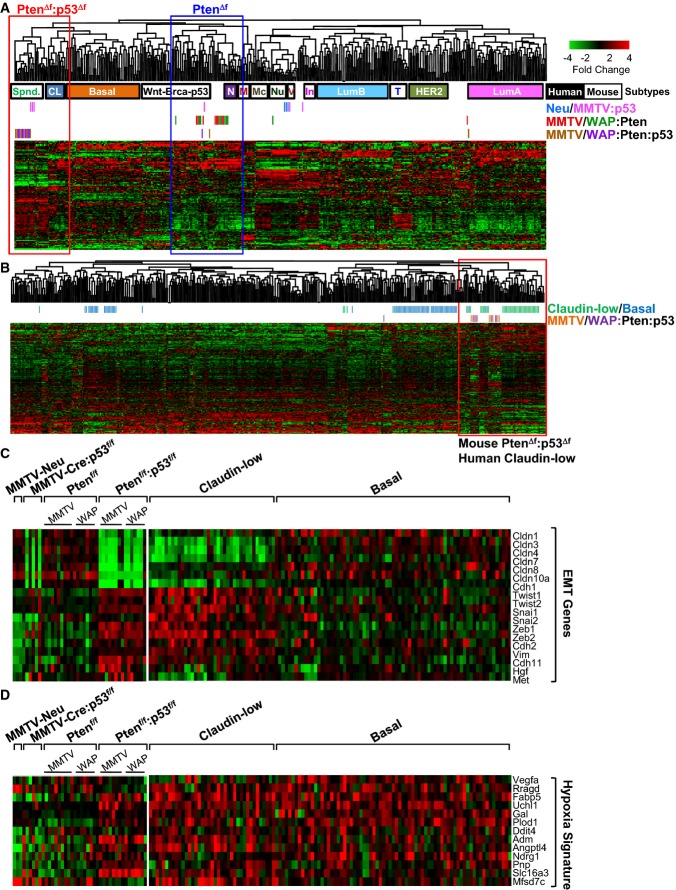
Pten/p53-deficient mammary tumors cluster with claudin-low TNBC Cluster analysis of Pten/p53-deficient mammary tumors using an intrinsic gene signature (Supplementary Table S1A and B) in comparison with human (solid boxes; basal, CL: claudin-low, LumA: luminal A, LumB: luminal B, HER2 and N: normal-like) and mouse (open boxes; Spnd.: spindle, M: mammary glands, Nu: MMTV-Neu, Mc: Myc-derived, V: MMTV-PyVT, In: MMTV-Int3, T: tag-derived, Wnt-Brca-p53: MMTV-Wnt1, Brca1-deficient, p53-deficient) BC samples.Cluster analysis of Pten/p53-deficient mammary tumors with human claudin-low (green) and basal-like (blue) BC using the Prat/Perou claudin-low signature. Mouse Pten/p53-deficient tumors clustered with human claudin-low—not with basal-like BC—on the far right. Non-claudin-low human and mouse tumors clustered together on the left.Expression of EMT genes in indicated mouse tumors and in human claudin-low versus basal-like BC.Expression of hypoxia signature genes in indicated mouse tumors and in human claudin-low versus basal-like BC.

To molecularly classify the Pten/p53-deficient tumors, we compared them to other mouse models and human BC subtypes using an extended intrinsic BC signature and unsupervised hierarchical clustering (Herschkowitz *et al*, 2007) (Supplementary Table S1A). Expression across platforms was combined and integrated using the distance weighted discrimination (DWD) algorithm (Benito *et al*, 2004). Three MMTV-Her2/Neu tumors were included as internal control. Cluster analysis grouped them with published MMTV-Her2/Neu tumors (Fig2A), thus validating our normalization process. Most (10/16) Pten^Δf^ tumors clustered with “normal”-like BCs. Importantly, the majority of MMTV-Cre:Pten^f/f^:p53^f/f^ and WAP-Cre:Pten^f/f^:p53^f/f^ tumors (12/15) clustered with mouse spindle-like mammary tumors and human claudin-low BC. In contrast, only half (3/6) of MMTV-Cre:p53^f/f^ tumors clustered with Pten^Δf^:p53^Δf^ tumors/claudin-low BC.

We next used a claudin-low signature developed by Prat and Perou to classify our Pten^Δf^:p53^Δf^ tumors with human BC samples (Prat *et al*, 2010) (Fig2B; Supplementary Table S1B). All but one Pten^Δf^:p53^Δf^ tumors clustered with claudin-low BC. Accordingly, expression of claudin 3, 4 and 7 was very low in 14 of 15 MMTV-Cre:Pten^f/f^:p53^f/f^ and WAP-Cre:Pten^f/f^:p53^f/f^ tumors (Fig2C). In contrast, only 3 of 6 MMTV-Cre:p53^f/f^ tumors expressed low levels of claudin genes. The mouse Pten^Δf^:p53^Δf^ tumors and most human claudin-low BC samples, but only 1 of 6 p53^Δf^ tumors, expressed high levels of the EMT inducers Twist1/2, Snail1/2 and Zeb1/2 (Fig2C; Supplementary Table S1C). A Basal-B/claudin-low BC cell signature (Blick *et al*, 2010) also resembled mouse Pten^Δf^:p53^Δf^ tumors better than p53^Δf^ tumors (Supplementary Fig S3A, Supplementary Table S1D).

TNBCs are known to be hypoxic (Tan *et al*, 2009). Using an hypoxic signature (Supplementary Table S1E) (Hu *et al*, 2009), we found that mouse Pten^Δf^:p53^Δf^ tumors and human claudin-low and basal-like BCs expressed high levels of hypoxia-related genes compared with most mouse p53^Δ^, Pten^Δf^ and Her2/Neu tumors (Fig2D). Finally, p53 induces several microRNAs (miRs) including miR200 that inhibit EMT by silencing expression of EMT inducers including Zeb2 (Gregory *et al*, 2008; Chang *et al*, 2011; Kim *et al*, 2011). In accordance, we found that expression of miR200a, miR200b, miR200c, miR429 and miR205 was significantly reduced in Pten^Δf^:p53^Δf^ versus Pten^Δf^ tumors (Supplementary Fig S3B and C, Supplementary Table S1F). In addition, Pten^Δf^:p53^Δf^ tumors exhibited significantly lower levels of p63 and Dicer, which regulate and process the miR200 family (Supplementary Fig S3D) (Su *et al*, 2010). Thus, combined inactivation of Pten and p53 induces mammary tumors with enhanced features of EMT and close resemblance to human claudin-low BC. As noted in the Introduction, mesenchymal-like cancer cells are observed in human breast tumors and may represent the cancer stem cell subpopulation. Thus, although Pten/p53 tumors exemplify an exaggerated form of this phenotype, insights derived from these models may prove valuable to targeting this most important fraction of tumor cells.

### 24 differentially expressed genes in WAP-Cre:Pten^f/f^:p53^f/f^ versus MMTV-Cre:Pten^f/f^:p53^f/f^ tumors can predict clinical outcome for claudin-low BC patients

**Figure 3:**
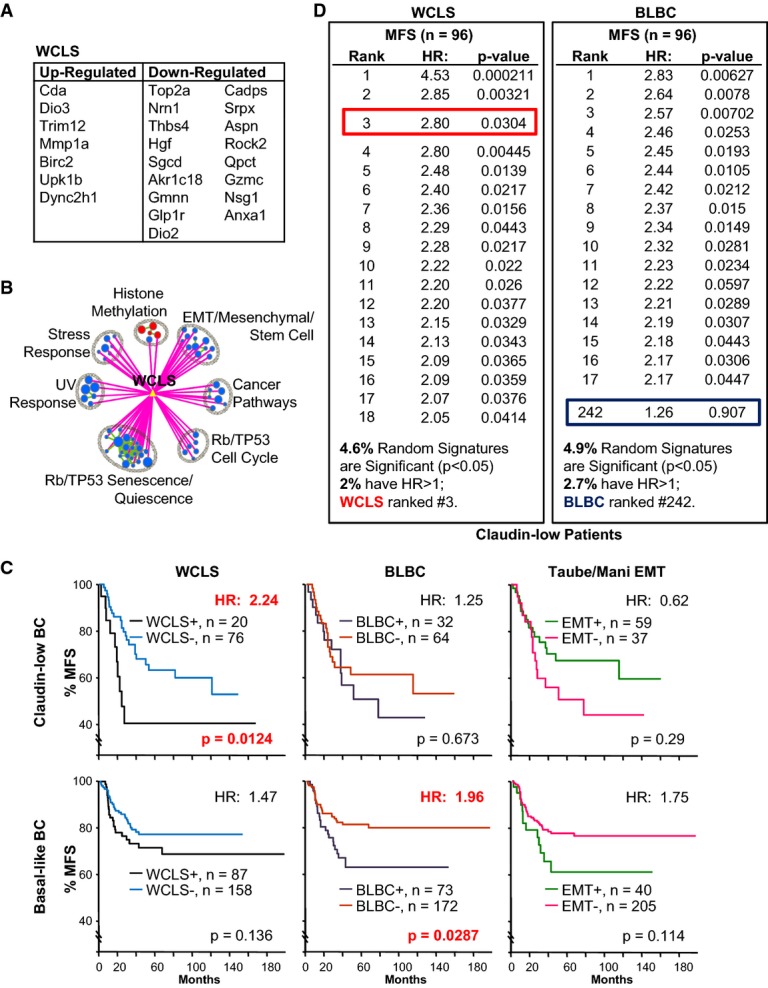
24 differentially regulated genes between WAP-Cre:Pten^f/f^:p53^f/f^ and MMTV-Cre:Pten^f/f^:p53^f/f^ tumors predict clinical outcome for claudin-low BC patients List of 24 genes that are significantly (FDR *q*-value <0.05) and differentially (> twofold) regulated between WAP-Cre:Pten^f/f^:p53^f/f^ and MMTV-Cre:Pten^f/f^:p53^f/f^ tumors, including 7 up-regulated and 17 down-regulated (Supplementary Table S1G).Selected pathways that are significantly associated with WCLS from GSEA analysis of WAP-Cre:Pten^f/f^:p53^f/f^ (red) versus MMTV-Cre:Pten^f/f^:p53^f/f^ (blue) tumors. Green lines connect overlapping pathways. Circle size corresponds to levels of enrichment and thickness of lines to degree of overlap. The full GSEA map and association with WCLS are shown in Supplementary Fig S4A.Kaplan–Meier metastasis-free survival (% MFS) curve for claudin-low and basal-like BC patients with WCLS, BLBC and the Taube/Mani EMT signature.Comparison of WCLS and BLBC relative to 1,000 random sets of signatures, generated from atmosphere background noise (random.org), with the same gene composition (7 up-regulated, 17 down-regulated for WCLS; 9 up-regulated, 5 down-regulated for BLBC) on claudin-low BC patients. The percentage of signatures with significant HR > 1.0 is listed at the bottom. WCLS ranked 3^rd^ for claudin-low BC, while BLBC ranked #242. For similar analysis on basal-like BC, see Supplementary Fig S4C.

We first probed for differences between WAP-Cre:Pten^f/f^:p53^f/f^ and MMTV-Cre:Pten^f/f^:p53^f/f^ tumors. To this end, we performed Global Gene Set Enrichment Analysis (GSEA) and visualized results using “Functional Enrichment Maps” (Merico *et al*, 2011). This analysis revealed several pathways that are differentially induced between WAP-Cre:Pten^f/f^ and MMTV-Cre:Pten^f/f^ tumors (Supplementary Fig S4A). Remarkably, direct comparison of mRNA levels from these two groups identified only 24 genes that were significantly (FDR *q*-value < 0.05) and differentially (> twofold) expressed (Fig3A). Seven of these genes were up-regulated (Cda, Dio3, Trim12, Mmp1a, Birc2, Upk1b, Dync2h1) and 17 down-regulated (Top2a, Nrn1, Thbs4, Hgf, Sgcd, Akr1c18, Gmnn, Glp1r, Dio2, Cadps, Srpx, Aspn, Rock2, Qpct, Gzmc, Nsg1, Anxa1). These 24 genes marked specific pathways in the GSEA, which were down-regulated in WAP-Cre:Pten^f/f^:p53^f/f^ relative to MMTV-Cre:Pten^f/f^:p53^f/f^ tumors including EMT/mesenchymal, UV and stress response as well as RB/p53-related cellular senescence (Fig3B and Supplementary Fig S4A).

We next asked whether the 24-gene set was predictive of clinical outcome using a cohort of 96 claudin-low BC patients with metastatic-free survival (MFS) data. Remarkably, this gene set, which we termed WAP-Cre Claudin-Low Signature (WCLS, Supplementary Table S1G), could stratify claudin-low patients into high and low risk groups with a hazard ratio of 2.24 (*P* = 0.0124; Fig3C). Comparing to a recently reported signature for Basal-Like Breast Cancer (BLBC) (Hallett *et al*, 2012), WCLS was specific for the claudin-low tumors, whereas BLBC was specific for basal-like BC with HR = 1.96 (*P* = 0.0287; Fig3C). Both signatures were not informative for HER2^+^, luminal A or luminal B BC (Supplementary Fig S4B).

To assess the possibility that WCLS is predictive by chance alone, we generated 1,000 random signatures with the same composition (i.e. 7 up-, 17 down-regulated genes) and analyzed their predictive power against the same patient cohort, as previously described (Liu *et al*, 2013). We found that 4.6% of the random signatures were significant (*P* < 0.05), of which 2% had HR > 1 (Fig3D). Importantly, WCLS ranked 3^rd^ with HR of 2.8 (*P* = 0.03), indicating that its prognostic power is statistically significant. In contrast, BLBC ranked 242 with insignificant *P*-value. For basal-like BC patients, BLBC, but not WCLS, ranked high (2^nd^) compared to 1,000 random signatures of similar composition (Supplementary Fig S4C).

The better prognosis of WCLS-negative versus WCLS-positive patients suggests that overt activation of EMT/mesenchymal pathways may improve outcome by blocking mesenchymal-to-epithelial transition (MET), which is required for metastatic growth at distal sites (Ocana *et al*, 2012; Tsai *et al*, 2012). In this case, an EMT signature should also not be associated with worse outcome. To test this prediction, we determined whether a core EMT/mesenchymal signature developed by Taube *et al* (2010) could predict clinical outcome, using the same claudin-low patient cohorts. We found that claudin-low patients expressing the Taube/Mani EMT signature did not show a poorer prognosis than signature-negative patients. In fact, there was a trend, albeit not statistically significant, toward better outcome (Fig3C). Taken together, our analysis shows that despite their similarity, there is a small number of genes that is significantly and differentially expressed between WAP-Cre:Pten^f/f^:p53^f/f^ and MMTV-Cre:Pten^f/f^:p53^f/f^ tumors and that this small gene set can predict clinical outcome for claudin-low BC patients.

### Unique and frequent tumor-initiating cells in Pten/p53-deficient claudin-low-like mammary tumors

**Figure 4:**
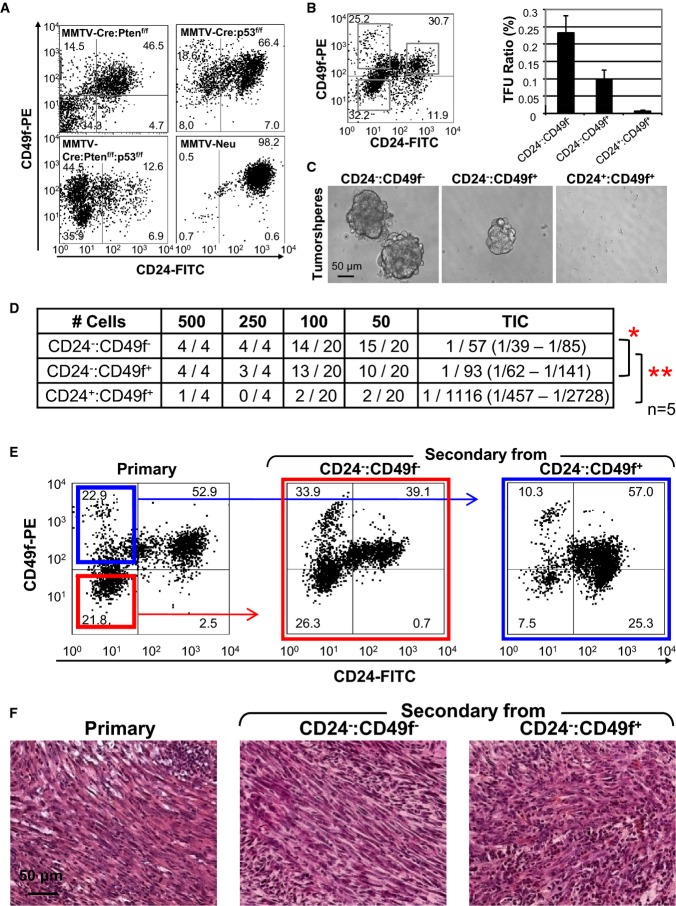
High frequency and unique tumor-initiating cells (TICs) in Pten/p53-deficient mic Flow cytometry profiles of indicated tumors with the CD24 and CD49f cell surface markers.Gating conditions used to sort MMTV-Cre:Pten^f/f^:p53^f/f^ tumor cells into CD24^−^:CD49f^−^, CD24^−^:CD49f^+^ and CD24^+^:CD49f^+^ fractions. CD24^−^:CD49f^−^ cells contained significantly higher tumorsphere-forming units (TFU) at a frequency of 0.23% (*P* < 0.00142, ANOVA with Tukey test for *post hoc*) compared with CD24^−^:CD49f^+^ or CD24^+^:CD49f^+^ fractions. The CD24^−^:CD49f^+^ fraction contained TFU at a frequency of 0.10% (*P* = 3.13 × 10^−5^, ANOVA with Tukey test for *post hoc*) relative to the CD24^+^:CD49f^+^ fraction.Representative images of tumorspheres from each fraction.TIC frequency in five MMTV-Cre:Pten^f/f^:p53^f/f^ tumors following cell sorting and orthotopic transplantation into Rag1^−/−^ females. There were too few cells in the CD24^+^:CD49f^−^ fraction for analysis. Significant differences in TIC frequencies were determined using L-Calc (Stemcell Technologies) and ANOVA. *P < 0.05; **P < 0.001.CD24/CD49f profiles of primary and secondary tumors from transplantation of CD24^−^:CD49f^+^ (blue) or CD24^−^:CD49f^−^ (red) MMTV-Cre:Pten^f/f^:p53^f/f^ tumor cells.Representative mesenchymal-like histology of a primary MMTV-Cre:Pten^f/f^:p53^f/f^ tumor and secondary tumors developed following transplantation of CD24^−^:CD49f^+^ or CD24^−^:CD49f^−^ cell fractions.

To determine the impact of combined Pten/p53 loss relative to p53 deletion alone, we analyzed cancer stem cell (CSC) populations in these tumors. CSCs represent a subset of tumor cells that is capable of sustaining tumorigenesis as well as giving rise to the tumor bulk, which is derived from CSCs but has lost its tumorigenic potential through epigenetic alterations (Kreso & Dick, 2014). CSCs are functionally defined as tumor-initiating cells (TICs) through their ability to seed new tumors following transplantation into recipient mice and to grow as spheres under non-adherent conditions (Liu *et al*, 2007; Deng *et al*, 2014). In the mouse, many mammary TICs are defined on the basis of CD49f (α6 integrin) and CD24 (a luminal marker) expression (Liu *et al*, 2007). Interestingly, in contrast to Pten^Δf^, p53^Δf^ and Her2/Neu tumor cells, which contained a prominent CD24^+^:CD49f^+^ double-positive cell fraction, most Pten^Δf^:p53^Δf^ mammary tumor cells expressed low levels of the luminal marker CD24 and were CD24^−^:CD49f^−^ or CD24^−^:CD49f^+^ (Fig4A and B). In accordance, Pten^Δf^:p53^Δf^ tumorsphere-forming units (TFUs), capable of growing into spheres when seeded onto ultra-low attachment plates in defined, serum-free media, were found predominantly in the CD24^−^:CD49f^−^ and CD24^−^:CD49f^+^ but not in the CD24^+^:CD49f^+^ double-positive fractions (Fig4B and C).

Sorted CD24^−^:CD49f^−^ or CD24^−^:CD49f^+^ Pten^Δf^:p53^Δf^ tumor cells also formed secondary tumors following orthotopic transplantation (*n* = 5, Fig4D). TIC frequency in these fractions was high (1/57 and 1/93, respectively) compared to 1/1,116 in the CD24^+^:CD49f^+^ fraction. The secondary tumors from these fractions recapitulated the heterogeneous flow cytometric profiles of, and were histologically indistinguishable from, the primary tumors from which they were derived (Fig4E and F). TICs in spindle-like p53^Δ^ mammary tumors were reported to be CD24^+^:CD49f^+^ (Herschkowitz *et al*, 2012). Thus, Pten deficiency cooperates with p53 mutation to accelerate tumorigenesis and promote more mesenchymal, CD24-negative TICs.

### Distinct signaling pathways in Pten/p53- versus p53-deficient claudin-low mammary tumors

**Figure 5:**
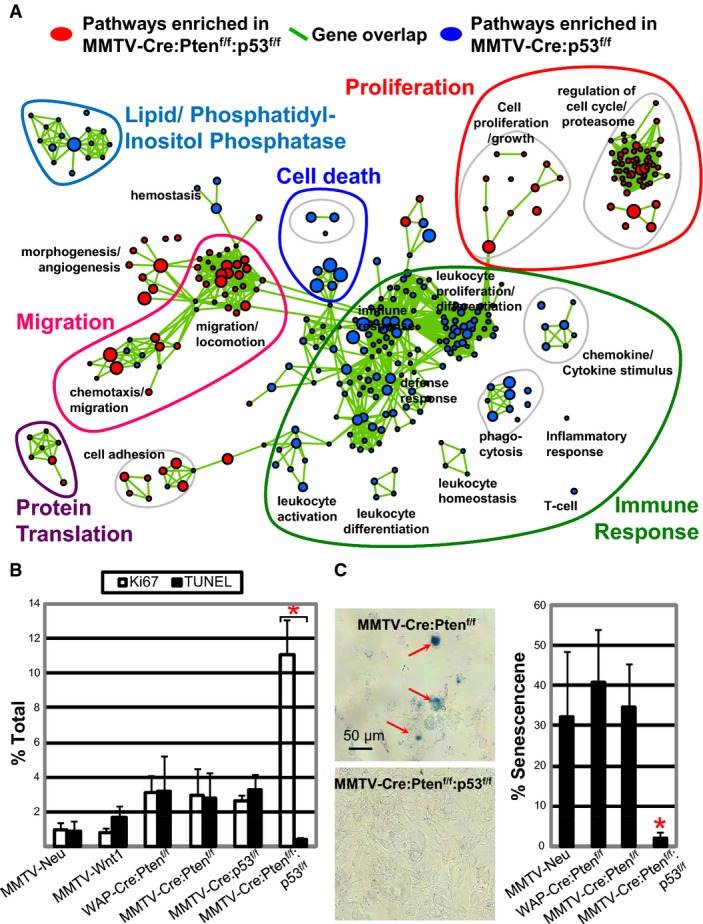
Pathway analysis of Pten/p53- versus p53-deficient claudin-low-like TNBC GSEA analysis showing selected pathways enriched in claudin-low-like MMTV-Cre:Pten^f/f^:p53^f/f^ (red) versus pathways enriched in MMTV-Cre:p53^f/f^ (blue) tumors. Green lines connect overlapping pathways. Proliferation, cell death, immune response, protein translation, and PI phosphatase pathways are highlighted; corresponding genes listed in Supplementary Table S1H–M. Full pathway analysis is shown in Supplementary Fig S5A.Cell proliferation (Ki67) and apoptosis (TUNEL) in indicated tumors. Representative staining and statistical analysis are shown in Supplementary Fig S5D and E.Senescence-associated β-Gal staining of indicated primary tumor cells plated on collagen-coated coverslips showing significantly lower level of senescence in MMTV-Cre:Pten^f/f^:p53^f/f^ tumor cells. Data information: *Significant difference comparing MMTV-Cre:Pten^f/f^:p53^f/f^ with MMTV-Neu, *P* = 0.012 (ANOVA with Tukey test for *post hoc*). Comparisons with other tumor models gave lower *P*-values.

To define transcriptional programs that distinguish Pten^Δf^:p53^Δf^ from p53^Δf^ tumors, we performed GSEA. Comparing claudin-low-like Pten^Δf^:p53^Δf^ versus claudin-low-like p53^Δf^ tumors, pathways associated with “migration/locomotion” and “cell proliferation” were up-regulated, whereas those associated with “immune response” and “cell death” were down-regulated (Fig5A, Supplementary Fig S5A, Supplementary Table S1H–M). Genes on the “lipid/phosphatidyl-inositol phosphatase” pathway were also altered. Additional comparisons of claudin-low plus non-claudin-low Pten^Δf^:p53^Δf^ and p53^Δf^ tumors, Pten^Δf^:p53^Δf^ versus Pten^Δf^, and p53^Δf^ versus Pten^Δf^ tumors are shown in Supplementary Materials and Methods and Supplementary Fig S5B–D.

To test whether Pten^Δf^:p53^Δf^ tumors exhibit increased cell proliferation and reduced apoptosis, as predicted from the pathway analysis, we stained tumor sections for cell proliferation using Ki67 and for cell death using TUNEL, which detects cleaved/nicked DNA, the hallmark of apoptosis. The ratio of cell proliferation to apoptosis was significantly higher in Pten^Δf^:p53^Δf^ tumors (*n* = 10) relative to Pten^Δf^, p53^Δf^, Wnt1 and Neu tumors (*n* = 3–5 for each; Fig5B). Supplementary Figure S5E and F shows examples of staining for these markers and statistical analysis. To assess cellular senescence, primary tumors were dissociated, lineage-depleted, seeded at similar densities onto collagen-coated cover slides and, 3 days later, stained for senescence-associated β-galactosidase activity (Debacq-Chainiaux *et al*, 2009). This revealed much reduced cellular senescence and increased cellularity in Pten^Δf^:p53^Δf^ compared to Pten^Δf^ or Her2/Neu tumor cells (Fig5C). Thus, relative to Pten^Δf^ or p53^Δf^ single mutant, Pten^Δf^:p53^Δf^ double-mutant claudin-low-like tumors exhibit multiple hallmarks of aggressive cancer.

### Low Pten-expression/p53-pathway activity identifies TNBC patients with poor clinical outcome

**Figure 6:**
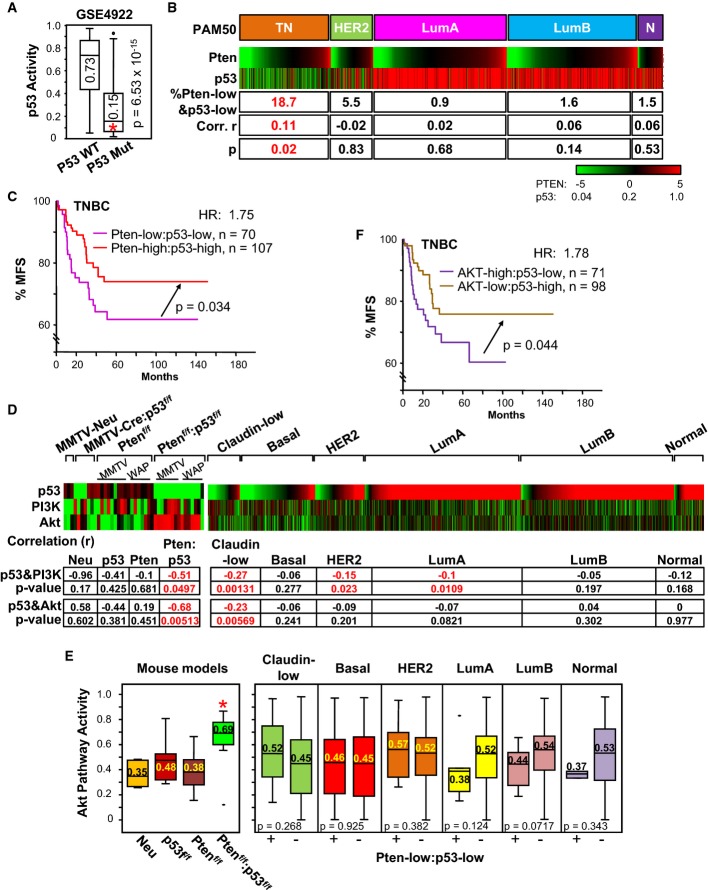
Correlation between Pten/p53 status and AKT pathway activity in BC and effect on clinical outcome Box plot for p53-pathway activity in p53 wild-type and p53 mutant tumors from GSE4922. Median value of p53-pathway activity for p53 mutant, 0.15, is lower than for wild-type tumors (0.73; *P* = 6.5 × 10^−15^, Mann–Whitney *U*-test).Pten gene expression and p53 pathway activity in human BC (PAM50 classification). Triple negative (TN) comprises claudin-low, basal-like and other subtypes. Number of tumors per group: TN, 471 (claudin low, 141; basal-like, 330); HER2^+^, 218; LumA, 687; LumB, 672; Normal, 131. Percentage of samples with low Pten expression and low p53 pathway activity (Pten-low and p53-low) is indicated for each subtype. Pearson's correlation (*r*) with statistical significance (*P*-value) for Pten gene expression versus p53 pathway activity is shown for indicated BC subtypes. Only TNBCs exhibited significantly higher % of samples with Pten-low and p53-low (*P* ≤ 5 × 10^−6^ relative the other subtypes) as well as positive correlation (*P* = 0.02).Kaplan–Meier metastasis-free survival (% MFS) analysis showing that TNBC patients with Pten-low:p53-low tumors have poorer prognosis than Pten-high:p53-high tumors (*P* = 0.034, Wilcoxon).p53, PI3K and AKT pathway activities in mouse models (left) and human BC subtypes (right). Note the high and significant correlations of p53 and PI3K as well as p53 and AKT pathways in mouse Pten/p53 tumors and human claudin-low TNBC.Box plot of AKT pathway activity in indicated mouse and human tumors. For mouse tumors, AKT pathway activity was significantly higher in Pten^f/f^:p53^f/f^ compared to p53^f/f^ tumors (*P = 0.04, Kruskal–Wallis). Comparisons with other models gave lower *P*-values. For human BC, AKT signaling was calculated for Pten/p53-low tumors (left boxes) versus all other tumors (right boxes). Note the high Akt pathway activity in mouse Pten/p53 tumors relative to other models (*P* < 0.04) and a trend toward increased Akt signaling in human Pten-low/p53-low versus other claudin-low tumors (*P* = 0.268). *P*-values (Kruskal–Wallis) are shown.Kaplan–Meier metastasis-free survival (% MFS) analysis showing that TNBC patients with AKT-high:p53-low tumors have poorer prognosis than AKT-low:p53-high tumors (*P* = 0.044, Wilcoxon).

To evaluate the effect of combined loss of Pten and p53 in TNBC, we used bioinformatics to identify Pten/p53-deficient patients with clinical data. Pten is often deregulated in BC through promoter methylation and microRNA-mediated silencing (Salmena *et al*, 2008; Koboldt *et al*, 2012), and its mRNA expression is the primary determinant of Pten protein levels in BC (Saal *et al*, 2007). We therefore assessed Pten RNA level from publicly available microarray expression data sets. For p53, we used a p53 pathway activity signature developed by Gatza *et al* (2010) (Supplementary Table S1N and O). We then took advantage of a BC cohort (GSE4922) with known p53 status to normalize pathway-activation values, using as a reference the median (0.15) of p53-mutant tumors (Fig6A). With these conditions, we determined Pten expression and p53 pathway activity for 2,179 patients including 471 TNBC, combined from 13 cohorts, six of which also had clinical information. Intrinsic BC subtypes were classified using PAM50 (Parker *et al*, 2009) (Supplementary Table S1P), and claudin-low TNBCs were identified using the Prat/Perou claudin-low signature (Supplementary Fig S6A). We found that 24.4% of TNBCs were Pten-low, 65.6% were p53-activity-low, and 18.7% were both Pten-low and p53-pathway-activity-low (Fig6B). This frequency of Pten-low/p53-low tumors in TNBC was significantly higher than in all other BC subtypes (*P* ≤ 5 × 10^−6^). In addition, only in TNBCs, there was a statistically significant correlation (0.11) between low Pten-expression and low p53-activity (*P* = 0.02; Fig6B). This positive yet low correlation is likely because in TNBC, p53 is often lost with other tumor suppressors, for example, INPP4B and RB, whereas Pten is often lost together with Brca1. Nevertheless, our results reveal that a substantial % of TNBC tumors (18.7%) is driven by combined loss of Pten and p53.

Importantly, patients harboring TNBC with low Pten expression and low p53-pathway activity had significantly worse metastatic-free survival (MFS) compared to those with normal expression of both tumor suppressors, with hazard ratio (HR) of 1.75 (*P* = 0.034; Fig6C). Tumors with only one of these tumor suppressors lost exhibited intermediate MFS curves that were not significantly different than those for Pten/p53-low or Pten/p53-normal tumors (not shown). Thus, both in mouse and in human, Pten/p53 deficiency leads to aggressive TNBC.

### AKT pathway activation occurs in mouse Pten^Δf^:p53^Δf^ tumors and a subset of human Pten/p53-deficient TNBC

We next assessed the impact of Pten/p53 deletion on PI3K/AKT signaling by calculating pathway activation for AKT, PI3K and p53 (Gatza *et al*, 2010). As expected, p53-pathway activity in mouse p53^Δf^ tumors was completely negative (Fig6D, p53 lane), thus validating the p53-pathway analysis. Interestingly, AKT pathway activity was only modestly elevated in the Pten-only or p53-deficient tumors relative to MMTV-Neu, but strongly induced in Pten/p53 double-mutant tumors (Fig6D and E), indicating that loss of Pten alone does not fully dysregulate the PI3K/AKT pathway. Indeed, a strong negative correlation between AKT- and p53-pathway activities was found in Pten/p53-deficient (−0.68; *P* = 0.005), but not in Pten or p53 single-mutant tumors. A negative correlation between the PI3K and p53 pathways was also seen in Pten/p53-deficient mammary tumors (−0.51; *P* = 0.049). Importantly, a box plot analysis revealed significantly elevated AKT signaling in mouse Pten/p53 claudin-low tumors (0.69) compared to p53^Δ^, Pten^Δf^ or Neu tumors (*P* < 0.04 by Kruskal–Wallis; Fig6E, Supplementary Table S1N and O). Consistent with this, we observed elevated Akt phosphorylation at Ser473 in Pten/p53-deficient tumors (Supplementary Fig S6B). Analysis of 15 additional signaling pathways (Gatza *et al*, 2010) revealed that MYC, E2F1 and β-catenin pathway activities were also induced in Pten/p53-deficient tumors as compared to other subtypes and that similar induction was seen in human TNBC (Supplementary Fig S6C, Supplementary Table S1N and O).

In human BC, we found a modest negative correlation between AKT versus p53 pathways, and PI3K versus p53 pathways (−0.27, *P* = 0.001; −0.23; *P* = 0.005, respectively) in claudin-low but not in basal-like TNBC (Fig6D). When examining AKT signaling, only Pten/p53-low claudin-low tumors showed a trend toward elevated AKT pathway activation (Fig6E). This trend was not sufficiently significant (*P* = 0.268), possibly because in human TNBC, AKT signaling is induced through both Pten-dependent and Pten-independent mechanisms. Together, this analysis demonstrates the existence of a subgroup of Pten/p53-deficient TNBC (18.7%) and that even within this subgroup, there is great variability in the level of AKT pathway activity, likely due to different cooperating oncogenic networks.

To determine whether AKT-pathway-high/p53-pathway-low activity could predict clinical outcome, we used the top 30% high AKT pathway activity as “cutoff” level. Patients with AKT-pathway-high/p53-pathway-low TNBCs had poorer prognosis than those with AKT-pathway-low/p53-pathway-high (HR = 1.78; *P* = 0.044; Fig6F). Thus, TNBC patients with high AKT signaling and/or low Pten expression plus p53 loss have poor clinical outcome and should be prioritized for aggressive or new therapy.

### Pten/p53-deficient claudin-low TNBC with elevated AKT signaling is susceptible to eEF2K inhibitors

**Figure 7:**
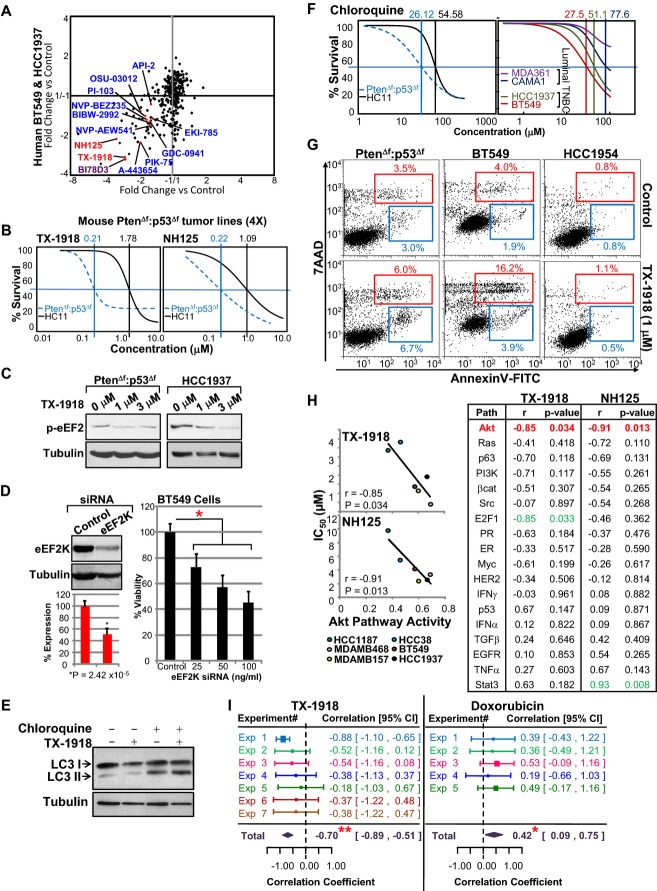
Kinome drug screen identifies eEF2K inhibitors as potential therapeutic targets for TNBC with elevated AKT signaling Kinase inhibitor screens on four independent primary MMTV-Cre:Pten^f/f^:p53^f/f^ tumor lines and two human Pten/p53 mutant BT549 and HCC1937 TNBC cell lines. Inhibitors for eEF2K (red), JNK (purple) and PI3K/AKT/mTOR (blue) are highlighted.Dose–response curves for mouse MMTV-Cre:Pten^f/f^:p53^f/f^ tumors versus immortalized HC11 epithelial cells treated with indicated eEF2K inhibitors.Inhibition of eEF2 phosphorylation by eEF2K inhibitor, TX-1918. Mouse or human Pten/p53-deficient tumor cells were serum-starved for 2 h, treated for 1 h with indicated concentrations of TX-1918 and immunoblotted with anti-Thr56 eEF2 antibody. Tubulin served as a loading control.BT549 cells were transfected with control (Dharmacon) or eEF2K siRNA at 25, 50 or 100 ng/ml. Two days later, cells were analyzed for eEF2K expression by Western blotting and for growth by MTT assay. *Significant difference comparing siRNA at 25 ng/ml with control, *P* = 0.00479 by ANOVA with Tukey test for *post hoc*. Comparisons at other concentrations gave lower *P*-values.Chloroquine but not TX-1918 suppresses autophagy/LC3-II accumulation.Dose–response curves for mouse MMTV-Cre:Pten^f/f^:p53^f/f^ tumor versus immortalized mouse epithelial HC11 cells and human TNBC (BT549, HCC1937) versus luminal (MDA361, CAMA1) cells treated with chloroquine.Levels of apoptosis in mouse Pten^Δf^:p53^Δf^, human BT549 and HER2^+^ HCC1954 cells after TX-1918 treatment as determined by Annexin V and 7AAD flow cytometry.Correlation analysis demonstrating that high AKT pathway activity but not 17 other signaling pathways sensitizes TNBC cells to eEF2K inhibitors TX-1918 and NH125.Meta-analysis demonstrating that high AKT pathway activity sensitizes TNBC cells to eEF2K inhibition (TX-1918). IC_50_ values for TX-1918 (*n* = 7) and control doxorubicin (*n* = 5) were determined in human TNBC cell lines (HCC38, HCC1937, BT549, MDAMB157, MDAMB436, MDAMB468). Correlation coefficient (*r*) of IC_50_ values with AKT pathway activities was calculated for each experiment by linear regression using meta-analysis, metaphor package in *r*: **r = −0.70, *P* < 0.0001 for TX-1918; *r = 0.42, *P* < 0.02 for doxorubicin.

To identify drugs that can target Pten/p53-deficient TNBCs with high AKT pathway activity, we performed a kinome drug screen (238 compounds targeting 154 different kinases; 3 μM; alamar blue assay) on four Pten^Δf^:p53^Δf^ tumor cultures, each established from a distinct MMTV-Cre:Pten^f/f^:p53^f/f^ mammary tumor. We also screened two human TNBC lines, HCC1937 and BT549, which harbor mutations in both tumor suppressors (Neve *et al*, 2006; Hollestelle *et al*, 2007; Blick *et al*, 2008). Top inhibitors from both screens were eukaryotic elongation factor-2 kinase (eEF2K: TX-1918; NH125) and c-Jun N-terminal kinase (JNK; BI78D3) (Fig7A, Supplementary Fig S7A). Multiple PI3K, AKT and PI3K/mTOR inhibitors such as PIK-75, A-443654 and NVP-BEZ235 were also identified (Fig7A, Supplementary Fig S7A), hence validating our screen, but they were not as efficient as the eEF2K or JNK inhibitors. Dose–response curves for TX-1918 and NH125 using MTT assays revealed IC_50_ of approximately 0.2 μM for mouse Pten^Δf^:p53^Δf^ tumors cells versus 1.1–1.8 μM for immortalized HC11 mammary epithelial cells (Fig7B). BI78D3 had IC_50_ of approximately 0.57 and 1.44 μM for Pten^Δf^:p53^Δf^ tumors and HC11 cells, respectively (Supplementary Fig S7B). Western blot analysis confirmed inhibition of eEF2 phosphorylation on Thr56 following treatment of mouse and human Pten/p53-mutant TNBC cells with the eEF2K inhibitor (Fig7C).

Interestingly, protein translation including eEF2 was one of the modulated pathways in Pten/p53 versus p53 tumors (brown circle, FigA, Supplementary Table S1M). eEF2K is required for growth and is elevated in many types of cancer (Silvera *et al*, 2010). Our non-biased screen of six different lines is the first to identify this kinase as one of the most potent targets for Pten/p53-deficient TNBC. Recently, eEF2K was shown to maintain survival of brain tumor cells under nutrient deprivation (Leprivier *et al*, 2013). In contrast, we identified eEF2K inhibitors under normal serum conditions. To further examine these results, we knocked down eEF2K using RNA interference (Dharmacon). This led to incomplete (∼50%) reduction in eEF2K protein expression, yet suppressed cell growth twofold under normal (nutrient abundant) conditions (*P* < 0.005) (Fig7D).

Both eEF2K and JNK are downstream targets of the PI3K pathway (Vivanco *et al*, 2007; Py *et al*, 2009; Hubner *et al*, 2012; Leprivier *et al*, 2013), and both have been implicated in autophagy (Zhang *et al*, 2008; Wu *et al*, 2009; Cheng *et al*, 2010). In accordance, suppression of autophagy by chloroquine (CQ), an inhibitor of autophagosome–lysosome fusion (Klionsky *et al*, 2012), revealed high autophagy flux (LC3-II accumulation) and efficient killing of both mouse and human Pten/p53-deficient tumor cells as compared to immortalized mammary epithelial cells or human luminal BC cells (Fig7E and F). However, pharmacological inhibition of eEF2K with or without CQ did not have a discernable effect on the LC3-II/LC3-I ratio under non-starving conditions (Fig7E), suggesting that eEF2K does not sustain growth by modulating autophagy in Pten/p53-deficient tumor cells. Instead, flow cytometry analysis for Annexin V, a marker for apoptosis, revealed that low concentrations of TX-1918 (1 μM) induced apoptotic cell death in both mouse and human TNBC cells but not in the HER2^+^ BC line HCC1954 (Fig7G).

Understanding the link between genetic alterations in cancer and response to therapy is crucial for stratifying patients for therapy. We therefore determined whether sensitivity of six human TNBC cell lines (BT549, HCC38, HCC1937, MDAMB157, MDAMB436, MDAMB468) to eEF2K inhibitors was proportional to any of the 18 pathway signatures defined by Gatza *et al* (2010) (Supplementary Table S1Q). Remarkably, only the AKT pathway signature significantly correlated with sensitivity of TNBC cells to TX-1918 and NH125 (*r* = −0.85, *P* = 0.034; *r* = −0.91, *P* = 0.01, respectively; Fig7H). We then extended the analysis to seven independent experiments, each in duplicates, and plotted IC_50_ against pathway activity in the different cell lines. Linear regression using meta-analysis revealed that the sensitivity of TNBC cells to TX-1918 had a correlation coefficient of −0.70 (*P* < 0.0001; Fig7I; Supplementary Fig S7C). In contrast, response to doxorubicin was reduced with increased AKT signaling (correlation coefficient = 0.42, *P* < 0.02). Thus, AKT pathway signaling may be used as a predictor for patient response to eEF2K inhibitors.

### eEF2K and JNK inhibitors suppress xenograft growth of Pten/p53-deficient claudin-low TNBC

**Figure 8:**
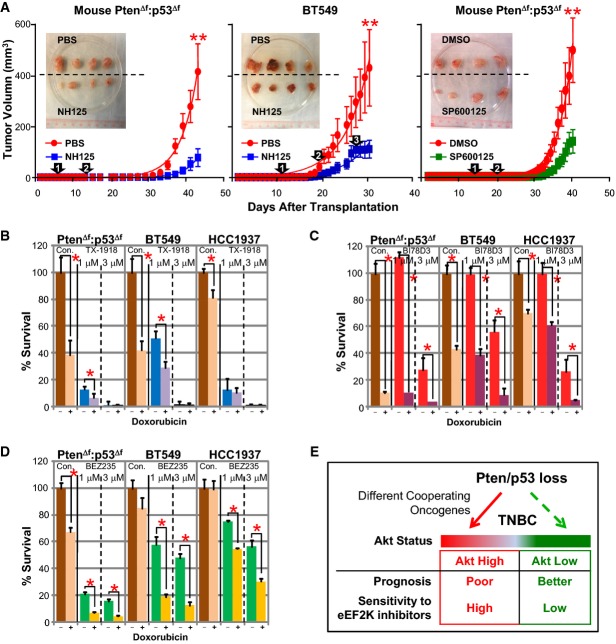
Anti-eEF2K or JNK monotherapy attenuates growth of Pten/p53-deficient TNBC xenografts Drug–response curves for mouse Pten^Δf^:p53^Δf^ and human BT549 tumor xenografts untreated or treated with eEF2K (NH125) or JNK (SP600125) inhibitors. Tumor cells were injected orthotopically into NOD/SCID mice. Mice (*n* = 9 per group) were injected with vehicle or NH125 (1 mg/kg) i.p. daily for 7 days (arrow #1), followed by every second day (arrow #2). For BT549 cells, mice were switched back to daily treatment for the final 4 days (arrow #3). SP600125 was administered at 60 mg/kg daily for 5 days (arrow #1) followed by 30 mg/kg daily (arrow #2). Inlets show 4 representative tumors of each group. Significant differences comparing control with treatment groups were determined by Graphpad Prism 5.0 with non-linear regression. **P < 0.0001.Response of Pten^Δf^:p53^Δf^ mouse and human tumor cells to TX-1918 +/− doxorubicin. Indicated cells were treated with low-dose doxorubicin (0.1 mg/ml, 170 nM) or 0 (Con), plus 1 or 3 μM TX-1918 for 3 days and analyzed by MTT assay. Additive effect of TX-1918 plus doxorubicin was observed (*P < 0.05, *t*-test).Response of Pten^Δf^:p53^Δf^ mouse and human cells to BI78D3 (JNK) +/− doxorubicin. Additive effect of BI78D3 plus doxorubicin was observed (*P < 0.05, *t*-test).Response of Pten^Δf^:p53^Δf^ mouse and human cells to NVP-BEZ235 (PI3K/mTOR) +/− doxorubicin. Synergistic effect of NVP-BEZ235 plus doxorubicin was observed (*P < 0.05, *t*-test).Our results suggest that combined Pten/p53 loss in human TNBCs induces a range of AKT pathway activation, depending on different cooperating oncogenic events, which affects prognosis and tumor response to anti-eEF2K therapy.

TX-1918 contains a reactive side chain that is predicted to interact with glutathione in the blood and diminish half-life. Thus, to determine the effect of eEF2K inhibition on xenograft growth, we used NH125, which is active *in vivo* (Arora *et al*, 2003). Following orthotopic injection of mouse or human Pten/p53-mutant TNBC cells, mice were treated with tolerable doses of NH125 (intraperitoneal, 1 mg/kg/daily for 1 week followed by 1 mg/kg every second day). Both mouse tumor volume and human tumor volume were significantly inhibited (*P* < 0.0001; Fig8A). For the treated human BT549 xenograft, relapse occurred before the end point. Switching back to daily treatment halted further growth (Fig8A; center, 3^rd^ arrow).

To test for the effect of JNK inhibitors on xenograft growth, we used SP600125, which unlike BI78D3 identified in our screen is stable *in vivo* (Ennis *et al*, 2005). Administration of this inhibitor also attenuated xenograft growth of mouse Pten/p53-mutant tumor cells *in vivo* (*P* < 0.0001; Fig8A, right).

Current treatment of TNBC patients involves cytotoxic drugs such as doxorubicin, which have serious adverse side effects. Targeted drugs that can cooperate with doxorubicin to kill TNBC may reduce toxicity and improve outcome. We therefore tested for cooperation between TX-1918 (eEF2K), BI78D3 (JNK) or NVP-BEZ235 (PI3K/mTOR) and doxorubicin. Using Compusyn software to assess level of synergy for drug combinations, we found that TX-1918 and BI78D3 had additive effects with doxorubicin (Fig8B–D). Notably, although similar trends were observed, responses to TX-1918 or BI78D3 alone or together with doxorubicin were stronger than to NVP-BEZ235. Together, these results suggest that while patients carrying TNBC with high AKT pathway activity have poor prognosis, they would benefit from anti-eEF2K (as well as anti-JNK) therapy in combination with doxorubicin, thus encouraging rapid development of effective eEF2K inhibitors (Fig8E).

## Discussion

TNBCs represent heterogeneous types of tumors that are highly aggressive and difficult to treat; metastatic disease is common and lethal. We found that the tumor suppressors Pten and p53 are lost together in over 18% of TNBC. Moreover, we showed that a subset of patients carrying Pten/p53-deficient TNBC have the worst prognosis compared to other TNBCs with normal level of these tumor suppressors. Using a kinome screen on primary mouse Pten^Δf^:p53^Δf^ tumors cells and Pten/p53 mutant TNBC lines, we identified eEF2K as well as JNK as potent therapeutic targets. Inhibitors of these targets were significantly more effective than PI3K, AKT or PI3K/mTOR antagonists, some of which are currently tested in the clinic on TNBC patients. Our results therefore identify both eEF2K and JNK as promising therapeutic targets for Pten/p53-deficient TNBC.

We disrupted Pten and/or p53 with two different deleter lines: WAP-Cre (which preferentially targets CD24^+^, pregnancy-identified luminal/alveolar progenitors) and MMTV-Cre^NLST^ (which targets both the CD49f^high^/CD24^+^ and CD24^+^ compartments) (Wagner *et al*, 2002; Jiang *et al*, 2010). Although tumor latency was shorter when Pten and p53 were deleted via WAP-Cre relative to MMTV-Cre, histology and cluster analysis revealed that tumors driven by these two promoter-Cre lines were indistinguishable. This is somewhat unexpected because BC subtypes have been linked to the cell-of-origin within the mammary epithelial cell hierarchy; claudin-low tumors are thought to arise from transformation of mammary stem cells, basal-like tumors from bi-potent/early luminal progenitors, HER2^+^ BC from more committed luminal progenitors, etc. (Lim *et al*, 2009; Prat & Perou, 2009). As WAP-Cre is expressed in pregnancy-identified CD24^+^ alveolar progenitors, our observation that WAP-Cre:Pten^f/f^ mice develop diverse types of mammary tumors whereas nearly all WAP-Cre:Pten^f/f^:p53^f/f^ (and MMTV-Cre:Pten^f/f^:p53^f/f^) mice give rise to claudin-low-like tumors supports a model whereby tumor subtype is dictated by both the cell-of-origin and the oncogenic/tumor suppressor networks that drive neoplastic transformation. Likewise, the fact that both WAP-Cre:Pten^f/f^ and MMTV-Cre:Pten^f/f^ mice gave rise to myoepithelioma, which is thought to originate from a myoepithelial cell of origin, suggests that combined deletion of Rb and p53 in luminal cells may induce dedifferentiation or transdifferentiation into myoepithelial cells. Two recent reports using other approaches or Cre drivers reached similar conclusions (Kim *et al*, 2014; Melchor *et al*, 2014).

Strikingly, despite the similarity between WAP-Cre:Pten^f/f^:p53^f/f^ and MMTV-Cre:Pten^f/f^:p53^f/f^ tumors, we identified 24 genes that were significantly and differentially expressed between the two models. This 24-gene set (WCLS) could stratify claudin-low patients into two groups with different clinical outcomes. WCLS marked low EMT/senescence, suggesting that tumors that originate from alveolar progenitors, as in WAP-Cre-driven tumors, are not locked in the EMT state as those driven by MMTV-Cre and are therefore more aggressive. Interestingly, in the prostate gland, a signature derived from luminal cells is also more predictive of poor patient outcome than a signature derived from tumors that originate from basal cells (Wang *et al*, 2013). Taken together, our results identify a novel predictor for claudin-low BC and support the idea that over-commitment to EMT diminishes metastasis.

Our genetic analysis of Pten^Δf^:p53^Δf^ tumors revealed several levels of cooperation between these two tumor suppressors. First, Pten/p53 tumors are induced faster than p53 or Pten single-mutant tumors and exhibit an increase in pathways associated with proliferation and motility and reduction in pathways associated with cell death and immune response. In addition, while only some p53-deficient tumors are sarcomatoid, nearly all Pten/p53-deficient tumors share this histology and cluster with human claudin-low TNBC. Finally, Pten/p53-deficient tumors had a prominent CD24^−^:CD49f^−^ fraction where most TICs reside, whereas both Pten- and p53-single knockout tumor cells are primarily CD24^+^:CD49f^+^. The absence of CD24 expression, a luminal marker, on Pten^Δf^:p53^Δf^ TICs underscores the highly mesenchymal nature of these tumors.

Second, we found that while AKT pathway activity was elevated in mouse Pten/p53-deficient mammary tumors, it was not consistently induced in human Pten/p53-low TNBC, likely because mutations in other components of the PI3K pathway, for example, Pik3ca, INPP4B, activate the pathway independently of Pten loss. Notably, Pten and PIK3CA are co-mutated in some human TNBCs (Yuan & Cantley, 2008; Lehmann *et al*, 2011), suggesting that mutations in more than one gene on the PI3K pathway may be required to fully activate the pathway, which is tightly autoregulated in normal cells (Cully *et al*, 2006). As a consequence, only a fraction of human Pten/p53-deficient TNBCs show high PI3K/AKT signaling and become sensitive to antagonists of this pathway. This model has direct implications for cancer therapy as it suggests that to guide therapy, patients should be screened for PI3K/AKT pathway activation or for multiple mutations along the pathway rather than for a single gene mutation (Janku *et al*, 2012; Rodon *et al*, 2013).

Our screen identified two eEF2K inhibitors as the most potent drugs against Pten/p53-deficient claudin-low TNBC cell lines. eEF2K is phosphorylated and inactivated by S6K1, downstream of mTORC1, leading to activation of eEF2 and mRNA translation elongation. eEF2K is also regulated by other signaling pathways including ERK and AMPK (Leprivier *et al*, 2013). Inhibition of eEF2K is thought to increase protein translation to unsustainable rate under nutrient deprivation, leading to cell demise. eEF2K has also been implicated in autophagy (Wu *et al*, 2009; Cheng *et al*, 2010). Interestingly, JNK is also linked to this process and, consistent with this, we found that Pten/p53-deficient tumor cells exhibit elevated autophagy flux and high sensitivity to the autophagosome–lysosome inhibitor CQ. However, inhibition of eEF2K (or JNK) did not affect autophagy under non-starving conditions. Instead, we found that eEF2K inhibition triggered apoptotic cell death even in nutrient-rich media through a mechanism that is not yet fully understood. Notably, TNBCs in general and as we show here Pten/p53-deficient tumors in particular are highly hypoxic. Hypoxia, similarly to nutrient deprivation, inhibits mTOR and protein translation in part by activating eEF2K and, therefore, eEF2K inhibitors may be exceptionally useful in treating hypoxic TNBC.

Available eEF2K inhibitors used herein have short half-life or off target effects (Arora *et al*, 2004). The essential role of eEF2K in Pten/p53-deficient TNBC (this study) and in brain cancer (Leprivier *et al*, 2013) should encourage development of specific and effective eEF2K inhibitors. These inhibitors may be used, as we showed here, as monotherapy, in combination with standard anthracycline therapy or with other drugs, such as a recently identified PLK4 inhibitor, which show strong anti-tumor activity against Pten-deficient BC (Mason *et al*, 2014). Moreover, we demonstrated that AKT pathway activity could predict response of TNBC to eEF2K inhibitors. Thus, development of a simple surrogate assay (e.g. immunostaining) for AKT pathway activation would simplify identification of TNBC patients who would benefit from anti-eEF2K-based therapy.

## Materials and Methods

### Animals

Mice used in this study were on mixed background (FvB, C57BL/6 and 129/sv): WAP-Cre mice were kindly received from Dr. Lothar Hennighausen, NIH, p53^f/f^ mice were obtained from the NCI Mouse Repository, and Pten^f/f^ mice were generated as described (Suzuki *et al*, 1998). For transplantation assays, we used immunocompromised Rag1^−/−^ females as recipients (JAX). Mice were housed in ventilated cages in our pathogen-free facility and monitored for mammary tumors as indicated in Figs1, 4 and 8. For this study, we used approximately 580 female mice for tumor analysis and transplantation assays. All experimental protocols were approved by the Toronto General Research Institute—UHN Animal Care Committee in accordance with the guidelines of the Canadian Council on Animal Care (AUP#10.50 and AUP#803).

### Bioinformatics

Microarray analysis with mouse tumor models was carried out using Affymetrix Mouse Gene 1.0 ST with 500 ng of total RNA isolated by double TRIzol extractions (Centre for Applied Genomics, Hospital for Sick Children, Toronto). Microarray data were normalized using RMA method via Partek software, and log2-transformed gene expression values were obtained.

For generating prognostic signature for claudin-low breast cancer (WCLS), ANOVA with FDR correction was performed between WAP-Cre:Pten^f/f^:p53^f/f^ and MMTV-Cre:Pten^f/f^:p53^f/f^ tumors to identify significantly (FDR *q*-value < 0.05) and differentially (> twofold) expressed genes. Kaplan–Meier and survival analysis were performed with PAST program (P.D. Ryan and Ø. Hammer, University of Oslo), and *P*-value was calculated by Wilcoxon method. Hazard ratios were obtained using the COX proportional hazards survival regression method. Heatmaps and dendrograms were generated by JAVA tree view.

Gene set enrichment analysis was performed using GSEA (Subramanian *et al*, 2005) with parameters set to 2,000 gene set permutations and gene sets size between 8 and 500. Gene sets were obtained from KEGG, MsigDB-c2, NCI, Biocarta, IOB, Netpath, Human Cyc, Reactome and the Gene Ontology (GO) databases.

**The paper explained**

**Problem**

Triple-negative breast cancer (TNBC) is a devastating subtype that affects approximately 10–20% of breast cancer patients. The tumor suppressors p53 and Pten are often inactivated in TNBC, but the consequences of combined mutations in these genes on tumorigenesis and response to therapy are largely unknown.

**Results**

We used Pten/p53-deficient mice and human TNBC cell lines to investigate how these two tumor suppressors cooperate to induce aggressive TNBC. We show that combined inactivation of Pten plus p53 via WAP-Cre or MMTV-Cre deleter lines induced claudin-low-like TNBC. We found 24 genes (WCLS) that are differentially and significantly expressed between MMTV-Cre:Pten^f/f^:p53^f/f^ and WAP-Cre:Pten^f/f^:p53^f/f^ double-mutant tumors and demonstrated that they can predict clinical outcome in claudin-low TNBC patients. Through non-biased kinome screens of mouse and human Pten/p53-deficient TNBC cells, we identified eEF2K as a potent inhibitor for TNBC with elevated AKT pathway activity.

**Impact**

WCLS-positive claudin-low TNBC patients should be prioritized for aggressive therapy. TNBC patients with elevated AKT pathway activity may benefit from anti-eEF2K therapy.

Exact *P*-values are shown except for ANOVA, where the highest *P*-value is given in the multi-group comparison, and for PRISM analysis, which does not provide specific *P*-values < 0.0001.

The microarray data from this publication have been submitted to the NCBI GEO database (http://www.ncbi.nlm.nih.gov/geo/) and assigned the identifier GSE39955.

### Kinase inhibitor screening, IC_50_ and MTT assay

238 compounds targeting 154 different kinases were screened using a Biomek FX liquid handler equipped with a pin tool for automated compound dispensing. Assays were carried out in a 384-well format with 300 cells/well.

### Xenograft assays

Pten:p53-mutant mouse (200,000 cells/injection) or human BT549 (1 million cells/injection) tumor cells were resuspended in 20 μl media/matrigel mixture (1:1) and injected into #4 mammary glands of NOD/SCID females (9 mice per group). Tumor-bearing mice were randomized and then treated intraperitoneally with NH125 (1 mg/kg, dissolved in PBS with 2% DMSO) or SP600125 (60 mg/kg and 30 mg/kg, dissolved in DMSO). Control mice were injected with vehicle alone at the same weight/volume ratio.

## Supporting Information

for this article is available online: http://embomolmed.embopress.org

Supplementary Table S1

Review Process File
