# Supplementary material for: Combined deletion of Pten and p53 in mammary epithelium accelerates triple-negative breast cancer with dependency on eEF2K
Source: EMBO Mol Med. 2014 Oct 20;6(12):1542–60. doi: 10.15252/emmm.201404402 (PMC4287974; doi:10.15252/emmm.201404402)
Supplement: Supplementary file 1 — Supplementary Information [file emmm0006-1542-sd1.pdf]

Combined Deletion of Pten and p53 in Mammary Epithelium Accelerates Triple-Negative Breast Cancer with Dependency on eEF2K

Jeff C. Liu, Veronique Voisin, Sharon Wang, Dong-Yu Wang, Robert A. Jones, Alessandro Datti, David Uehling, Rima Al-awar, Sean E. Egan, Gary D. Bader, Ming Tsao, Tak W. Mak, Eldad Zacksenhaus

Corresponding author: Eldad Zacksenhaus, University Health Network

Review timeline:

Submission date:	04 July 2014
Editorial Decision:	21 August 2014
Revision received:	05 September 2014
Editorial Decision:	16 September 2014
Revision received:	23 September 2014
Accepted:	25 September 2014

Transaction Report:

(Note: With the exception of the correction of typographical or spelling errors that could be a source of ambiguity, letters and reports are not edited. The original formatting of letters and referee reports may not be reflected in this compilation.)

Editor: Céline Carret

1st Editorial Decision 21 August 2014

Thank you for the submission of your manuscript to EMBO Molecular Medicine. We have now heard back from the three referees whom we asked to evaluate your manuscript.

You will see that all three referees are supportive of publication, however not in this form and they all suggest refocusing the paper in terms of writing (for a general audience) but also streamlining in terms of data presentation. In addition both referees 1 and 3 suggest few discussion points to strengthen the manuscript.

Given these evaluations, I would like to give you the opportunity to revise your manuscript, with the understanding that the referee concerns must be fully addressed and that acceptance of the manuscript may entail a second round of review.

Revised manuscripts should be submitted within three months of a request for revision; they will otherwise be treated as new submissions, except under exceptional circumstances in which a short extension is obtained from the editor. Also, the length of the revised manuscript may not exceed 60,000 characters (including spaces). You may consider including any peripheral data (but not methods in their entirety) in the form of Supplementary information.

I look forward to seeing a revised form of your manuscript as soon as possible.

***** Reviewer's comments *****

Referee #1 (Remarks):

The authors presented a study of exploring the signaling and functional consequences of a dual p53/PI3K deletion.

Overall, this was a well constructed study that was clearly written for an expert in the field. It was a little dense at times, I might suggest the authors "put some signposts" along the way to show how all the pieces in the text fit. Also, this figures are a bit "data rich". I suggest the authors only present the *most* critical data and relegate some of the nice, but spurious data to the supplemental.

1) L9, abstract - please revise the sentence "...Kinome screens identified eukaryotic elongation factor-2 kinase (eEF2K) as more potent than PI3K/AKT/mTOR inhibitors for both mouse and human Pten/p53-deficient TNBC cells. " More potent for what?

2) What is the phosphorylation state of AKT and 4EBPx, and level of eIF4E in the cell types most sensitive to eE2FK inhibition? AKT can be phosphorylated at least two positions (S473 and T308) and 4EBPx at many more. I'm surprised by the differential sensitivity to eE2FK inhibitors, and would have expected the same sensitivity to mTOR inhibition if this was a deregulated translation axis. Thus, it would be nice to understand what exactly AKT is doing, quantifying its phosphorylation state would give a clue as to how signals are integrated.

3) Activated AKT is also known to have pro-survival functions, e.g. Cell, Vol. 96, 857-868, March 19, 1999 (among many others). What is the specific mechanism at play in this model leading to enhanced survival? These could also represent potential targets, as an untargeted systemic eE2FK inhibitor seems to be non-starter clinically?

Referee #2 (Comments on Novelty/Model System):

Overall the manuscript presents interesting well carried out work, with medical implications, potentially high. The medical impact score should probably be between medium and high. In light of the large body of work presented I hesitated to ask for major additional experiments. I believe the paper can be suitable for publication if reorganized and central less informative sections are removed.

Referee #2 (Remarks):

This study by the Zacksenhaus group explores the effects of perturbation of the p53 and PTEN tumor suppressors on breast cancer development. p53 is mutated in the vast majority of the aggressive triple negative tumors, while PTEN is often silenced in them. The authors conduct simultaneous knockout of these two genes using two breast Cre lines (WAP and MMTV) and find that this significantly decreases the tumor latency compared to the deletion of each single gene, and changes the histological and molecular profile of the tumors. All genotypes give rise to heterogeneous tumor types, but while PTEN deletion primarily generates adenomyoepitheliomas (a subtype containing a prominent myoepithelial component), co-deletion of p53 gives rise mostly to tumors of a sarcomatoid/mesenchymal subtype. This is interesting, and implicates p53 in control of tumor identity, and suggests collaborative effects of the two genes. The authors follow this up with a broad bioinformatic study of the tumors of the different genotypes, identification of a cancer stem cell population, and analysis of p53 and PTEN expression and activity in human breast cancers. In the last section of the manuscript, they conduct a drug screen to identify kinase inhibitors that repress the growth of p53+PTEN-deficient tumor cells, and identify two inhibitors targeting eEF2K and one targeting JNK. They go on to show that inhibitors of these kinases decrease tumor growth in vivo and enhance tumor cell sensitivity to doxorubicin.

Overall the study is informative and well executed. Triple negative breast tumors pose a significant challenge in the clinic and therefore the elucidation of the molecular mechanisms that control and maintain these tumors is an important task. Similarly, it is clear that p53 and the PI3K pathway are the most important genetic drivers in breast cancer, and there are gaps in the understanding of their roles. The manuscript presents important information derived from these complex mouse models and presents a wealth of data, including very high-level execution of the histological, molecular and informatic studies. Its conclusions are of interest and relevance to the field. The discovery of eEF2K and JNK inhibitors as potential means to target triple negative breast cancers with high AKT activity is novel, and, potentially, of great importance.

Overall the study is informative and well executed. Triple negative breast tumors pose a significant challenge in the clinic and therefore the elucidation of the molecular mechanisms that control and maintain these tumors is an important task. Similarly, it is clear that p53 and the PI3K pathway are the most important genetic drivers in breast cancer, and there are gaps in the understanding of their roles, especially in dictating tumor subtype. The manuscript presents important information derived from these complex mouse models and presents a wealth of data, including high-level execution of the histological, molecular and informatic studies. Its conclusions are of interest to the field. The discovery of eEF2K and JNK inhibitors as potential means to target triple negative breast cancers with high AKT activity is novel, and, of potential importance.

The main weaknesses of the paper are two. The first is that the tumors developing in mice do not nicely mimic common human breast adenocarcinomas, including the great majority of triple-negative (or basal-like) tumors. Adenomyoepithelomas are very rare in human patients, and the mesenchymal tumors that appear in the PTEN+p53 KO mice are similar to metaplasias or claudin-low tumors, which are also rare in human patients. This problem raises the question of how much of the conclusions apply to the human disease, and the manuscript title may therefore be somewhat misleading. Nevertheless the authors go a long way in linking their findings with large panels of human breast cancer expression profiles and clinical outcomes, and clearly delineate the connection between the mouse and human findings. Furthermore, there is much evidence indicating that mesenchymal-like cancer cells (EMT'd) are present in human breast tumors and may represent a subpopulation of cancer stem cells and/or cells with increased metastatic capacity. In this light, even if the mouse models represent an exaggerated form of this phenomenon, the insights derived from theses models may prove valuable to the targeting of this most important fraction of tumor cells.

The second weakness is that the authors included in the manuscript several distinct research directions, which are poorly linked to each other. Furthermore, some of these present findings that are less informative than others. The consequence is that the manuscript lacks focus, and it is burdened by sections that read as digressions, particularly in Figures 3 and 6.

While the manuscript is well-written, an obvious problem is that figure legends throughout the paper do not provide the minimal information necessary for understanding the data: graph axes are unexplained, analyses are not clarified, and basic aspects of the experiments are lacking. This must be corrected.

Specific Comments:

Figure 1: Probably the most important finding here is the effect of p53 deletion on tumor histology, specifically the preferential generation of metaplastic/sarcomatoid tumors in PTEN/p53 KO mice. This finding is consistent with a recent paper by the Smalley group in which deletion of PTEN with and without p53 was conducted in the luminal mammary epithelium using a different Cre driver, in which lack of p53 also resulted in increased tendency towards metaplasias. The authors describe the tumors generated in the PTEN-only KO mice as "differentiated adenocarcinoma (adenomyoepithlioma)"; this is histopatologically inaccurate and should be corrected: differentiated adenocarcinomas are the common malignant breast carcinoma type, but adenomyoepitheliomas are not included in this definition: they are rare and mostly benign tumors that are distinct in the fact that they contain a large myoepithelial-like component. The authors should clarify what they refer to as "differentiated adenocarcinoma" and what is included under this definition.

Previous work suggests that adenomyoepithliomas can arise from a myoepithelial cell of origin;

while the MMTV-cre could potentially inactivate p53/PTEN in some myoepithelial cells, the WAP-cre is luminal specific. The fact that luminal cells can give rise to such tumors is interesting, and suggests that PTEN loss induces dedifferentiation towards the basal lineage.

Specific notes: The vimentin stains in Figures S1/2 appear to stain only stroma and not tumor cells; K5 and ER stains in S2 look like normal ducts. K5 is not a mesenchymal marker (p6) but a basal epithelial marker. There is no explanation in the legend of the PCR reaction shown in panel 1e.

Figure 2: Legends to the presented clustering analyses are lacking. The authors should clarify which tumors are included in the top sections, especially in B, and which of the lines/bars indicate their own mouse profiles. Which genes are shown in these panels? Do the blue and green bars in B indicate classified human tumors, and why are these tumors not clustered together? Why are the PTEN and p53 single KO tumors not included in B? These two charts (A and B) seem quite redundant, and the authors should consider placing one of them in supplementary data.

Figure 3. The authors go to great lengths to compare and analyze the expression profiles of WAP-cre versus MMTV-cre PTEN+p53 KO tumors, as the former show a decreased latency. The motivation for comparing the two Cre lines, however, is unclear, since the tumor histological and molecular profiles are very similar, and the basis for the different latency could be purely technical (e.g. recombination efficiencies). They identify 24 genes that show differential expression between these tumors, and argue that these represent a prognostic signature for Claudin low tumors. While this may be true, in light of the rarity of Claudin-low tumors in the clinic, it is not clear how useful this signature can be. 24 genes are also too small a number to derive insightful pathway/biological information. Overall this section seems to detract from the paper by diverting attention to a side issue of poor biological interest. The authors should consider removing it.

Legend for panel 3B is not sufficient to explain the data (e.g. what is the meaning of the colors and size of circles?).

Figure 4: The authors analyze the identity of tumor initiating and sphere forming cell in the tumor models. There have been several different reports of markers for breast cancer stem cells identified in mouse models, and overall these often do not match the human marker profile. Nevertheless the data here are well executed and are of interest, indicating that a CD24- population, whose numbers are increased in the p53+PTEN KO mice, contains increased tumor initiation potential. Low CD24 probably indicates loss of epithelial identity as is seen in cD24 breast CSCs.

Are the tumors compared in A of different subtypes?

FACS plots in A,B,E should include the % of cells in each quadrant.

Figure 5: Here the authors present a pathway analysis of the gene expression profiles of the PTEN KO versus PTEN+P53 KO mice. This is a direct continuation of the data in Figure 2, and for logical continuity should be placed in that Figure or directly after it. The legends offer minimal information and interpretation is quite confusing - there are blue and red circles as well as pathways, there is no clarification of the meaning of line length, circle sizes, the nature of the connections and more. Compared are only claudin-low versus claudin low tumors; additional tumor types are included in analysis in the supplementary data, however that data is so minimally annotated that it is not clear it contains informative findings.

Figure 6. In this figure the authors turn to human gene expression data in the attempt to better characterize the subset of p53 and PTEN silenced tumors. While this is important, this section is data heavy, and some of the analyses do not appear informative enough to justifying their inclusion.

In panels B, C the authors try to point to TNBC with low p53 and pten and show they have poor prognosis. Why is metastasis free survival shown and not overall survival? Panel B is somewhat difficult to follow: Claudin low tumors are not indicated, even though they are discussed as potentially generated by p53/PTEN loss; because the way the tumors are ordered one cannot appreciate the size of the PTEN-low p53-low TN population. The conclusion that p53 and PTEN loss are cooperative seems unwarranted on the basis of the observed correlation of 0.11, but this does not mean that co-deletion of the genes has functional consequences. Do tumors with low p53

and PTEN show preferential expression of mesenchymal traits/genes/EMT or other aspects found in the mice? This would link the human tumors to the previous findings in mice.

The data in panels D,E are not very informative and in my opinion detract from the paper. It is not clear that the Akt activity signature derived from the Nevins paper is of use here (it is not activated in the PTEN KO mice in which clearly Akt is active), and it is difficult to draw strong conclusions as to the manner by which p53 affects Akt activity in human tumors. The manuscript would probably be better off without these panels and their lengthy description.

Figures 7-8: Identification of the eEF2K and JNK inhibitors in this screen and their effectiveness are highly interesting findings. Legend to Panel 7A does not provide any information as to the parameters tested in this screen, and there is no indication as to the meaning of the axes in this graph. Was the tumor from which the cells in the screen, and the lines transplanted in the mice of a metaplatic nature? The contention that sensitivity to eEF2K inhibition is dependent on Akt activation would be supported by a demonstration of the effects of Akt/Pi3K inhibition on sensitivity to these drugs in the PTEN mutant cells, or of introduction of activated Akt (myr-Akt) to a PTEN proficient cell line (HCC1954).

Referee #3 (Comments on Novelty/Model System):

Genetically engineered mouse model has been used to study human cancers mainly because of the inability to study gene function in human.

Referee #3 (Remarks):

In this manuscript, Liu et al. characterized the in vivo models of breast cancers driven by combination of Pten and p53 mutations and identified eEF2K as a potential therapeutic target against triple-negative breast cancer type.

1. The authors found that the inactivating mutations of Pten and p53, found in a subset of human breast cancer patients, are an important determinant for the development of mouse tumor resembling triple-negative breast cancer. Although several recent papers (Kim et al, 2014; PMID: 24531711) (Melchor et al, 2014; PMID: 24615332) described models and phenotypes similar to those in this study, the findings here were more comprehensive and, unlike the other studies, provided insight into therapy. Perhaps the authors would discuss the papers to stress the points of their study.

2. The authors identified a specific gene set through comparative gene expression analysis of the Pten:p53 breast cancer models and demonstrated the ability of the gene set to predict the clinical outcomes of patients with certain subtypes especially claudin-low breast cancer. These patients generally have worse prognosis. Intriguingly, however, the authors argue that the EMT/mesenchymal feature in the gene set does not predict poor outcome, although the gene set was derived from the Pten:p53 tumors with enhanced feature of EMT and close resemblance to human claudin-low cancer. This does not appear to be in line with the prevailing idea of EMT/mesenchymal features contributing to chemoresistance and poor prognosis. The authors might want to discuss this point further.

3. The authors also identified the transcriptional program that underlies the phenotypes of Pten:p53 tumors, which includes signaling pathways for tumorigenesis. Furthermore, using a kinome screening, they identified eEF2K as a potential target to inhibit the growth of Pten:p53-deficient breast cancer. This is an interesting discovery that may provide a basis for patient (genotype)-specific chemotherapy. This part of the study would have been even better if the authors tested the effects of combining eEF2K inhibitor with doxorubicin using in vivo experiments using the mouse model or xenograft of human breast cancer cell lines. If the results show a synergistic effect on tumor inhibition, that would greatly increase the impact of the manuscript.

Overall, this study is well done and comprehensive, using a genetically engineered mouse model and extensive bioinformatics analyses of mouse and human tumors as well as a large amount of patient data. The major conclusions are generally well supported by the experimental data. Given that the findings of this study are also seen in other types of cancer, this manuscript will be of broad interest.I hope that the authors will address the major points made above and the minor points below.

Minor comments

Page 1, line 10, the authors probably meant ' Kinome screens identified inhibitors of eukaryotic ... (eIF2K) ...'

Page 4, line 3, the authors probably meant 'when p53 loss is not actionable'

Page 5, line 2 (bottom), conventional designation of the mouse genotype may be Pten / instead of Pten f, which could better describe heterozygote.

Page 6, line 3, K5 (keratin 5) is not a mesenchyal marker.

Page 7, second paragraph, the point of the first sentence is unclear. The phenotypes of WAP-Cre/Pten and WAP-Cre/Pten/p53 were not similar phenotype. If the first sentence is the rational for the following experiment, based on the point of the title and the following description of the results, the sentence should read "Although WAP-Cre directed deletion of Pten:p53 ... , the WAP-Cre Pten:53 exhibited shorter latency compared with MMTC-Cre Pten:p53. (The changes are underlined.) If this is not what the authors intended, this part needs to be clarified.

Page 8, last paragraph, the point of the sentence "The favorable..." is unclear.

The authors need to clarify why EMT core signature does not predict the outcome while WCLS is in line with EMT/mesenchymal. Need a little more explanation.

Page 13, why the others are not efficient? Not efficient for growth inhibition? The authors need to explain a little more their criteria to determine what the drugs are 'efficient'. Why are the other drugs close to the chosen ones being excluded? There are a few more drugs that appear to work very well but are not explained at all.

Page 13, line 2 (bottom), the authors probably meant " in many types of cancer"

Page 14, line 1, "Notably, we identified eEF2K inhibitors (?) under non-starving conditions." It is unclear what this means.

Do these 6 lines indicate 4 mouse primary cultures and 2 human cell lines? The authors need to describe the results more clearly.

Page 16, the authors wrote "We estimated that the tumor suppressors... lost...in over 18% of these tumors." Is this statement based on any references or any data presented? The authors need to clarify.

Page 16, line 8, the authors probably meant "Indeed, the inhibitors of these targets...."

1st Revision - authors' response 05 September 2014

Referee #1 (Remarks):

The authors presented a study of exploring the signalling and functional consequences of a dual p53/PI3K deletion.

*Overall, this was a well constructed study that was clearly written for an expert in the field. It was a little dense at times, I might suggest the authors "put some signposts" along the way to show how all the pieces in the text fit. Also, this figures are a bit "data rich". I suggest the authors only present the *most* critical data and relegate some of the nice, but spurious data to the supplemental.*

Critique:

1) L9, abstract - please revise the sentence "...Kinome screens identified eukaryotic elongation factor-2 kinase (eEF2K) as more potent than PI3K/AKT/mTOR inhibitors for both mouse and human Pten/p53-deficient TNBC cells. " More potent for what?

Response:

We thank the reviewer for the kind words.

The noted sentence has been modified to " Kinome screens identified eukaryotic elongation factor-2 kinase (eEF2K) **inhibitors** as more potent than PI3K/AKT/mTOR inhibitors….".

Critique:
2) What is the phosphorylation state of AKT and 4EBPx, and level of eIF4E in the cell types most sensitive to eE2FK inhibition? AKT can be phosphorylated at least two positions (S473 and T308) and 4EBPx at many more. I'm surprised by the differential sensitivity to eE2FK inhibitors, and would have expected the same sensitivity to mTOR inhibition if this was a deregulated translation axis. Thus, it would be nice to understand what exactly AKT is doing, quantifying its phosphorylation state would give a clue as to how signals are integrated.

Response:

We agree with the reviewer that this is indeed an important issue. In addition to AKT activation by bioinformatic analysis (Figure 6), we have now included a Western blot for S473 phospho-Akt, showing that mouse PTEN:p53-deficient tumour cells have higher Akt phosphorylation than other models. This immune-blot is presented in the **revised supplementary Figure 6B**.

Critique:
3) Activated AKT is also known to have pro-survival functions, e.g. Cell, Vol. 96, 857-868, March 19, 1999 (among many others). What is the specific mechanism at play in this model leading to enhanced survival? These could also represent potential targets, as an untargeted systemic eE2FK inhibitor seems to be non-starter clinically?

Response:
In this manuscript, we showed that AKT pathway activity varies among different TNBC lines and that it is a critical determinant in tumour response to eEF2K inhibitors. We agree with the reviewer that additional analysis is important to identify the exact mechanism that leads to elevated AKT pathway activity, enhanced survival and increased sensitivity to eEF2K inhibitor. In a future study, we intend to perform a more comprehensive analysis (exome sequencing, RNA-seq, proteomics), which is beyond the scope of this manuscript, to correlate response to eEF2K inhibitors with genomic alterations and identify the basis for AKT pathway activation in different tumours. Regarding eEF2K therapy – we think that combination of potent eEF2K inhibitors with targeted delivery such as nanoparticles would enable clinical utility of this drug.

Referee #2 (Comments on Novelty/Model System):

Overall the manuscript presents interesting well carried out work, with medical implications, potentially high. The medical impact score should probably be between medium and high. In light of

the large body of work presented I hesitated to ask for major additional experiments. I believe the paper can be suitable for publication if reorganized and central less informative sections are removed.

Referee #2 (Remarks):

This study by the Zacksenhaus group explores the effects of perturbation of the p53 and PTEN tumour suppressors on breast cancer development. p53 is mutated in the vast majority of the aggressive triple negative tumours, while PTEN is often silenced in them. The authors conduct simultaneous knockout of these two genes using two breast Cre lines (WAP and MMTV) and find that this significantly decreases the tumour latency compared to the deletion of each single gene, and changes the histological and molecular profile of the tumours. All genotypes give rise to heterogeneous tumour types, but while PTEN deletion primarily generates adenomyoepitheliomas (a subtype containing a prominent myoepithelial component), co-deletion of p53 gives rise mostly to tumours of a sarcomatoid/mesenchymal subtype. This is interesting, and implicates p53 in control of tumour identity, and suggests collaborative effects of the two genes. The authors follow this up with a broad bioinformatic study of the tumours of the different genotypes, identification of a cancer stem cell population, and analysis of p53 and PTEN expression and activity in human breast cancers. In the last section of the manuscript, they conduct a drug screen to identify kinase inhibitors that repress the growth of p53+PTEN-deficient tumour cells, and identify two inhibitors targeting eEF2K and one targeting JNK. They go on to show that inhibitors of these kinases decrease tumour growth in vivo and enhance tumour cell sensitivity to doxorubicin.

Overall the study is informative and well executed. Triple negative breast tumours pose a significant challenge in the clinic and therefore the elucidation of the molecular mechanisms that control and maintain these tumours is an important task. Similarly, it is clear that p53 and the PI3K pathway are the most important genetic drivers in breast cancer, and there are gaps in the understanding of their roles. The manuscript presents important information derived from these complex mouse models and presents a wealth of data, including very high-level execution of the histological, molecular and informatic studies. Its conclusions are of interest and relevance to the field. The discovery of eEF2K and JNK inhibitors as potential means to target triple negative breast cancers with high AKT activity is novel, and, potentially, of great importance.

Overall the study is informative and well executed. Triple negative breast tumours pose a significant challenge in the clinic and therefore the elucidation of the molecular mechanisms that control and maintain these tumours is an important task. Similarly, it is clear that p53 and the PI3K pathway are the most important genetic drivers in breast cancer, and there are gaps in the understanding of their roles, especially in dictating tumour subtype. The manuscript presents important information derived from these complex mouse models and presents a wealth of data, including high-level execution of the histological, molecular and informatic studies. Its conclusions are of interest to the field. The discovery of eEF2K and JNK inhibitors as potential means to target triple negative breast cancers with high AKT activity is novel, and, of potential importance.

Critique:

The main weaknesses of the paper are two. The first is that the tumours developing in mice do not nicely mimic common human breast adenocarcinomas, including the great majority of triple-negative (or basal-like) tumours. Adenomyoepithelomas are very rare in human patients, and the mesenchymal tumours that appear in the PTEN+p53 KO mice are similar to metaplasias or claudin-low tumours, which are also rare in human patients. This problem raises the question of how much of the conclusions apply to the human disease, and the manuscript title may therefore be somewhat misleading. Nevertheless the authors go a long way in linking their findings with large panels of human breast cancer expression profiles and clinical outcomes, and clearly delineate the connection between the mouse and human findings. Furthermore, there is much evidence indicating that mesenchymal-like cancer cells (EMT'd) are present in human breast tumours and may represent a subpopulation of cancer stem cells and/or cells with increased metastatic capacity. In this light, even if the mouse models represent an exaggerated form of this phenomenon, the insights derived from theses models may prove valuable to the targeting of this most important fraction of tumour cells.

Response:

We thank the reviewer for his/her excellent summation of the benefits of this mouse model (underlined statement). Indeed, we took the liberty to add a similar statement to the manuscript on page 7.

"As noted in the Introduction, mesenchymal-like cancer cells are present in human breast tumours and may represent a subpopulation of cancer stem cells. Thus, although these mouse models represent an exaggerated form of this phenotype, the insights derived from these models may prove valuable to the targeting of this most important fraction of tumour cells."

Critique:

The second weakness is that the authors included in the manuscript several distinct research directions, which are poorly linked to each other. Furthermore, some of these present findings that are less informative than others. The consequence is that the manuscript lacks focus, and it is burdened by sections that read as digressions, particularly in Figures 3 and 6.

Response:

We agree that Figure 3 – the development of a prognostic signature for claudin-low BC – may seem as deviation. However, it is a very insightful observation that shows that although WAP-Cre:Pten$^{f/f}$:p53$^{f/f}$ and MMTV-Cre:Pten$^{f/f}$:p53$^{f/f}$ tumours appear histology indistinguishable, and cluster very closely together, they differentially express 24 gene that, remarkably, can predict clinical outcome for claudin-low TNBC. This analysis therefore uncovers otherwise unappreciated and important differences between the 2 models. We respectfully think that the benefits of presenting these exciting results outweigh the small detour.

We also agree that Figure 6 is a bit difficult to digest. However, it is a critical aspect of the analysis. We made several to modifications to simplify the text. We also added the data on the % of tumours with low Pten/low p53 activity (**revised Fig. 6 panel B**), which is very informative. Overall the figure shows that TNBC contains a subset (~19%) of low-Pten/low-p53 tumours, and that AKT pathway activity is elevated in mouse Pten/p53 deficient models as well as in a subset of, but not all, TNBC. These observations set the stage for the subsequent analysis (Fig. 7), in which we show a positive correlation between tumour response to eEF2K inhibitors and AKT-pathway activity. Figure 6 informs us that the latter varies from tumour to tumour, even within Pten-low/p53-activity-low tumours, and should be determined by pathway activity analysis, not by identifying Pten loss or PI3K mutations.

Critique:

While the manuscript is well-written, an obvious problem is that figure legends throughout the paper do not provide the minimal information necessary for understanding the data: graph axes are unexplained, analyses are not clarified, and basic aspects of the experiments are lacking. This must be corrected.

Response:

We kept the legend succinct to meet space requirements. We have however reviewed all legends and added additional information and clarify the text when appropriate.

Specific Comments:

Critique:

Figure 1: Probably the most important finding here is the effect of p53 deletion on tumour histology, specifically the preferential generation of metaplastic/sarcomatoid tumours in PTEN/p53 KO mice. This finding is consistent with a recent paper by the Smalley group in which deletion of PTEN with and without p53 was conducted in the luminal mammary epithelium using a different Cre driver, in which lack of p53 also resulted in increased tendency towards metaplasias. The authors describe the tumours generated in the PTEN-only KO mice as "differentiated adenocarcinoma (adenomyoepithlioma)"; this is histopatologically inaccurate and should be corrected: differentiated adenocarcinomas are the common malignant breast carcinoma type, but adenomyoepitheliomas are

not included in this definition: they are rare and mostly benign tumours that are distinct in the fact that they contain a large myoepithelial-like component. The authors should clarify what they refer to as "differentiated adenocarcinoma" and what is included under this definition.

Response:

We thank the reviewer for noting this oversight on our part. These tumours are myoepitheliom. We have corrected this in Figure 1 and text.

Critique:

Previous work suggests that adenomyoepithliomas can arise from a myoepithelial cell of origin; while the MMTV-cre could potentially inactivate p53/PTEN in some myoepithelial cells, the WAP-cre is luminal specific. The fact that luminal cells can give rise to such tumours is interesting, and suggests that PTEN loss induces dedifferentiation towards the basal lineage.

Response:

We agree and have now included this point in the revised discussion (page 17/18).

Critique:

Specific notes: The vimentin stains in Figures S1/2 appear to stain only stroma and not tumour cells; K5 and ER stains in S2 look like normal ducts. K5 is not a mesenchymal marker (p6) but a basal epithelial marker. There is no explanation in the legend of the PCR reaction shown in panel 1e.

Response:

In Pten-single deletion tumours, vimentin staining was seen mostly in the stromal compartment (Figure S1A). In PTEN:p53 double deletion models, vimentin staining appears more in the tumour compartment but not in fully mesenchymal areas (Figure S2A). The predominant and more definitive marker for the EMT (PTEN:p53 model) is desmin (Figure S2B). We have corrected the label for K5 as basal epithelial marker. While ductal-like structures are present, they are different from normal ducts with their compact acinar formation (Figure 1B, S1A) or with their lumen filled with cells (Figure S2A).

We have now included an explanation for PCR in the legend.

Critique:

Figure 2: Legends to the presented clustering analyses are lacking. The authors should clarify which tumours are included in the top sections, especially in B, and which of the lines/bars indicate their own mouse profiles. Which genes are shown in these panels? Do the blue and green bars in B indicate classified human tumours, and why are these tumours not clustered together? Why are the PTEN and p53 single KO tumours not included in B? These two charts (A and B) seem quite redundant, and the authors should consider placing one of them in supplementary data.

Response:

We have clarified the cluster analysis in Figure 2B in the legend. Classified human claudin-low (green) and basal-like (blue) BC were used to compare with mouse Pten/p53 tumours. Other tumour subtypes, both human and mouse (single PTEN and Neu models) are included in the analysis but because Prat's signature is specific for claudin-low BC, the non-claudin-low tumours cluster together at the left.

Figure 2A shows the clustering of Pten/p53 tumours with Basal BC (TN), which include basal-like, claudin-low and other subtypes - while Figure 2B shows the specific claudin-low subtype of BC. The genes used for cluster analysis are listed in supplementary table S1A and S1B.

Critique:

Figure 3. The authors go to great lengths to compare and analyse the expression profiles of WAP-cre versus MMTV-cre PTEN+p53 KO tumours, as the former show a decreased latency. The motivation for comparing the two Cre lines, however, is unclear, since the tumour histological and molecular profiles are very similar, and the basis for the different latency could be purely technical (e.g. recombination efficiencies). They identify 24 genes that show differential expression between these tumours, and argue that these represent a prognostic signature for Claudin low tumours. While this may be true, in light of the rarity of Claudin-low tumours in the clinic, it is not clear how useful this signature can be. 24 genes are also too small a number to derive insightful pathway/biological information. Overall this section seems to detract from the paper by diverting attention to a side issue of poor biological interest. The authors should consider removing it.

Response:

We agree that WAP-cre and MMTV-cre PTEN+p53 KO tumours are very similar, and this is in part why our observation that they differentially express a small number of genes that have a prognostic power is so exciting. Indeed, our results suggest that other studies comparing similar tumour histology may benefit from checking for small differences in gene expression, which may promote certain signalling pathways. As we noted in the Discussion, Wang, et al, 2013 in Nat. Cell Bio., showed similar phenomena after comparing luminal-derived vs basal –derived prostate tumours.

We note that WCLS is the only signature to date with the ability to stratify claudin-low patients. WCLS should be useful for identifying claudin-low patients with most aggressive tumours, which should be given the most aggressive or experimental therapy, and in understanding mechanisms of claudin-low cancer progression.

Our side-by-side analysis with the recently developed signature for basal-like BC suggests that these two signatures can predict outcome for most types of TNBC.

The pathways associated with the 24 genes of WCLS (Fig. 3B) are based on a full GSEA analysis (not just the 24 genes!), comparing WAP-Cre:PTEN:p53 and MMTV-Cre:PTEN:p53. The full GSEA analysis has now been added to **revised Figure S4A**.

Critique:

Legend for panel 3B is not sufficient to explain the data (e.g. what is the meaning of the colours and size of circles?).

Response:

The additional information has been added to the legend of Figure 3B.

Critique:

Figure 4: The authors analyse the identity of tumour initiating and sphere forming cell in the tumour models. There have been several different reports of markers for breast cancer stem cells identified in mouse models, and overall these often do not match the human marker profile. Nevertheless the data here are well executed and are of interest, indicating that a CD24- population, whose numbers are increased in the p53+PTEN KO mice, contains increased tumour initiation potential. Low CD24 probably indicates loss of epithelial identity as is seen in cD24 breast CSCs.

Are the tumours compared in A of different subtypes?

Response:

Yes - they are from Pten and p53 single deletions, and from MMTV-Neu transgenic mice. Their corresponding subtypes are shown in Figure 2A.

Critique:

FACS plots in A,B,E should include the % of cells in each quadrant.

Response:

% of cells in each quadrant has now been included in **revised Figure 4**.

Critique:

Figure 5: Here the authors present a pathway analysis of the gene expression profiles of the PTEN KO versus PTEN+P53 KO mice. This is a direct continuation of the data in Figure 2, and for logical continuity should be placed in that Figure or directly after it. The legends offer minimal information and interpretation is quite confusing - there are blue and red circles as well as pathways, there is no clarification of the meaning of line length, circle sizes, the nature of the connections and more. Compared are only claudin-low versus claudin low tumours; additional tumour types are included in analysis in the supplementary data, however that data is so minimally annotated that it is not clear it contains informative findings.

Response:

We note that figure 5 shows Pten/p53 vs p53 (not Pten). For a comparison to Pten tumours – see supplemental Fig. S5C. We maintain the original order of figures as Fig. 3 compares MMTV-Cre:Pten/p53 to WAP-Cre:Pten/p53, which is something we focus on in the beginning of the manuscript. We later compared Pten/p53 to p53 tumours (Figures 4 and 5 as well as 6) to show the impact of Pten loss on p53-deficient TNBC. As requested, we have now included additional information/annotation in the legends to clarify the figures.

Critique:

Figure 6. In this figure the authors turn to human gene expression data in the attempt to better characterize the subset of p53 and PTEN silenced tumours. While this is important, this section is data heavy, and some of the analyses do not appear informative enough to justifying their inclusion.

Response:

We have addressed the importance of Fig. 6 and the changes we made to simplify the text in our response to the second Critique by this reviewer (see above).

Critique:

In panels B, C the authors try to point to TNBC with low p53 and pten and show they have poor prognosis. Why is metastasis free survival shown and not overall survival? Panel B is somewhat difficult to follow: Claudin low tumours are not indicated, even though they are discussed as potentially generated by p53/PTEN loss; because the way the tumours are ordered one cannot appreciate the size of the PTEN-low p53-low TN population. The conclusion that p53 and PTEN loss are cooperative seems unwarranted on the basis of the observed correlation of 0.11, but this does not mean that co-deletion of the genes has functional consequences. Do tumours with low p53 and PTEN show preferential expression of mesenchymal traits/genes/EMT or other aspects found in the mice? This would link the human tumours to the previous findings in mice.

Response:

There is a limited number of publically available cohorts with calculated p53-pathway activity, which is required for this analysis; we only have sufficient number of such cohorts (p53-pathway activity) with Metastasis-free survival data to perform Kaplan-Meier analysis.

The % of Pten-low/p53-low tumours is significantly higher in TNBC (18.7%) than in other subtypes. This information has now been added to the **revised Figure 6B**.

We agree that the correlation between low Pten-expression and low p53-pathway activity is not very strong in TNBC. However, this is the only statistically significant correlation we observed in BC. Moreover, the highest % of such tumours (18.7%) is found in TNBC (newly added data; Fig. 6B). Thus, our analysis suggests that p53 and pten are frequently lost in TNBC (18.7%), and should be specifically targeted for therapy. However, p53 is lost together with other genes in TNBC (e.g. RB1,

INPP4B), whereas Pten is often lost with Braca1, hence the modest correlation between Pten and p53. We noted this in the revised text on page 12 (tio).

Figs. 2B, 2C and Fig. 4 show that the low p53 and PTEN tumours do preferentially express mesenchymal traits/genes/EMT.

Critique:

The data in panels D,E are not very informative and in my opinion detract from the paper. It is not clear that the Akt activity signature derived from the Nevins paper is of use here (it is not activated in the PTEN KO mice in which clearly Akt is active), and it is difficult to draw strong conclusions as to the manner by which p53 affects Akt activity in human tumours. The manuscript would probably be better off without these panels and their lengthy description.

Response:

We think that Figure 6D and E demonstrate the importance of Akt signalling in Pten-low and p53-deficient claudin-low BC. These results are crucial for understanding the biology of these tumours, they form the basis for the testing the prognosis of tumours with high AKT/low p53, and explain the results from our drug screen as eEF2K is a downstream effector of Akt signalling. We do agree that the data were a bit condensed and we tried to simplify the results and clarify the text.

We also agree that AKT signalling is not dramatically elevated in Pten-deficient tumours. We note that the hit map shows relative expression – not absolute. We added the following statement to address this observation (p12): "Interestingly, AKT pathway activity was only modestly elevated in the Pten-only or p53-deficient tumours relative to MMTV-Neu, but strongly induced in Pten/p53-double mutant tumours (**Figure 6D-E**), indicating that loss of Pten-alone does not fully dysregulate the PI3K/AKT pathway. "

Critique:

Figures 7-8: Identification of the eEF2K and JNK inhibitors in this screen and their effectiveness are highly interesting findings. Legend to Panel 7A does not provide any information as to the parameters tested in this screen, and there is no indication as to the meaning of the axes in this graph. Was the tumour from which the cells in the screen, and the lines transplanted in the mice of a metaplatic nature? The contention that sensitivity to eEF2K inhibition is dependent on Akt activation would be supported by a demonstration of the effects of Akt/Pi3K inhibition on sensitivity to these drugs in the PTEN mutant cells, or of introduction of activated Akt (myr-Akt) to a PTEN proficient cell line (HCC1954).

Response:

The screen was performed in vitro using 4 independent cell lines generated from Mouse Pten/p53 tumours and 2 human cell lines BT549 and HCC1937. The assay we used for the drug screen was based Alamar blue staining as noted in the revised text (p13). The labels of the axis are fold changes vs control and we have clarify this in the revised figure (7A).

Both mouse and human cell lines are highly mesenchymal, give rise to mesenchymal tumours when transplanted, and show little metaplastic transformation. We agree with the reviewer that additional experiments such as introducing activated Akt (myr-Akt) to test for increased sensitivity would further corroborate our results. We intend to perform such analysis as well as a more comprehensive exome sequencing, RNA-seq and proteomics, which are beyond the scope of this manuscript, to correlate response to eEF2K inhibitors with genomic alterations and identify the basis for AKT pathway activation.

Referee #3 (Comments on Novelty/Model System):

Genetically engineered mouse model has been used to study human cancers mainly because of the inability to study gene function in human.

Referee #3 (Remarks):

In this manuscript, Liu et al. characterized the in vivo models of breast cancers driven by combination of Pten and p53 mutations and identified eEF2K as a potential therapeutic target against triple-negative breast cancer type.

Critique:

1. The authors found that the inactivating mutations of Pten and p53, found in a subset of human breast cancer patients, are an important determinant for the development of mouse tumour resembling triple-negative breast cancer. Although several recent papers (Kim et al, 2014; PMID: 24531711) (Melchor et al, 2014; PMID: 24615332) described models and phenotypes similar to those in this study, the findings here were more comprehensive and, unlike the other studies, provided insight into therapy. Perhaps the authors would discuss the papers to stress the points of their study.

Response:

We appreciate the comments and have included a statement about these recent studies including references in the Discussion (page 18)

Critique:

2. The authors identified a specific gene set through comparative gene expression analysis of the Pten:p53 breast cancer models and demonstrated the ability of the gene set to predict the clinical outcomes of patients with certain subtypes especially claudin-low breast cancer. These patients generally have worse prognosis. Intriguingly, however, the authors argue that the EMT/mesenchymal feature in the gene set does not predict poor outcome, although the gene set was derived from the Pten:p53 tumours with enhanced feature of EMT and close resemblance to human claudin-low cancer. This does not appear to be in line with the prevailing idea of EMT/mesenchymal features contributing to chemoresistance and poor prognosis. The authors might want to discuss this point further.

Response:

From our data it appears that Pten-deficiency in a luminal cell background will lead to more aggressive cancer. Other than tumour dissemination, which is promoted by EMT, the final step in the metastatic cascade involves colonization at distal site, which involves reversal of this process through mesenchymal-to-epithelial transition (MET). In Results (pp8-9) and Discussion (p18), we proposed that tumours that originate from luminal cells might undergo MET more readily and therefore form macrometastases more readily leading to poor MFS. We note that both MMTV-Cre- and WAP-Cre-derived Pten/p53 tumours are mesenchymal. Our hypothesis however is that over-commitment to (non-reversal) EMT interferes with macro-metastasis. We referenced several reports that support this concept (p8-9). We note that the 24-gene signature likely detects additional processes, not only EMT, which contribute to its predictive power.

Critique:

3. The authors also identified the transcriptional program that underlies the phenotypes of Pten:p53 tumours, which includes signalling pathways for tumourigenesis. Furthermore, using a kinome screening, they identified eEF2K as a potential target to inhibit the growth of Pten:p53-deficient breast cancer. This is an interesting discovery that may provide a basis for patient (genotype)-specific chemotherapy. This part of the study would have been even better if the authors tested the effects of combining eEF2K inhibitor with doxorubicin using in vivo experiments using the mouse model or xenograft of human breast cancer cell lines. If the results show a synergistic effect on

tumour inhibition, that would greatly increase the impact of the manuscript.

Response:

We agree that demonstrating cooperation of eEF2k inhibitors with doxorubicin in vivo would further strengthen our results. However, we feel that the most critical next-step, which we are actively pursuing, is to generate new, potent and highly specific eEF2K inhibitors that can be taken to the clinic. Once developed, we will perform drug combinations with doxorubicin and other drugs currently used to treat TNBC.

Critique:
Overall, this study is well done and comprehensive, using a genetically engineered mouse model and extensive bioinformatics analyses of mouse and human tumours as well as a large amount of patient data. The major conclusions are generally well supported by the experimental data. Given that the findings of this study are also seen in other types of cancer, this manuscript will be of broad interest. I hope that the authors will address the major points made above and the minor points below.

Response:
We thank the reviewer for the suggestions above and below, which further improve the manuscript.

Minor comments

Critique:
Page 1, line 10, the authors probably meant ' Kinome screens identified inhibitors of eukaryotic ... (eIF2K) ...'

Response:

Sentence corrected

Critique:
Page 4, line 3, the authors probably meant 'when p53 loss is not actionable'

Response:

Sentence corrected

Critique:
Page 5, line 2 (bottom), conventional designation of the mouse genotype may be Pten∆/∆ instead of Pten∆f, which could better describe heterozygote.

Response:

Unfortunately, the suggested designation of these lines was lost - but the reviewer seems to suggest we label the line $Pten^{f/f}$ rather than $Pten^{f}$. The latter refers to the allele; the former to the homozygous state.

Critique:
Page 6, line 3, K5 (keratin 5) is not a mesenchyal marker.

Response:

We agree and corrected the sentence accordingly.

Critique:
Page 7, second paragraph, the point of the first sentence is unclear. The phenotypes of WAP-Cre/Pten and WAP-Cre/Pten/p53 were not similar phenotype. If the first sentence is the rational for the following experiment, based on the point of the title and the following description of the results, the sentence should read "Although WAP-Cre directed deletion of Pten:p53 ... , the WAP-Cre Pten:53 exhibited shorter latency compared with MMTC-Cre Pten:p53. (The changes are underlined.) If this is not what the authors intended, this part needs to be clarified.

Response:

We thank the reviewer for this comment. We meant what we wrote. However, we have now changed this sentence to "We first asked whether any differences exist between WAP-Cre:Pten$^{f/f}$:p53$^{f/f}$ and MMTV-Cre:Pten$^{f/f}$:p53$^{f/f}$ tumours."

Critique:
Page 8, last paragraph, the point of the sentence "The favourable..." is unclear.

Response:

We changed "favourable" to "better".

Critique:
The authors need to clarify why EMT core signature does not predict the outcome while WCLS is in line with EMT/mesenchymal. Need a little more explanation.

Response:

Our results suggest that WCLS is predictive because it identifies tumours that are more similar to WAP-Cre driven tumours, which targets luminal cells, and that over-commitment to EMT locks tumour cells in a mesenchymal state that prevents MET, which is required for metastatic colonization (Ocana et al, 2012; Tsai et al, 2012). We discuss this model in page 9 top and Discussion.

Critique:
Page 13, why the others are not efficient? Not efficient for growth inhibition? The authors need to explain a little more their criteria to determine what the drugs are 'efficient'. Why are the other drugs close to the chosen ones being excluded? There are a few more drugs that appear to work very well but are not explained at all.

Response:

We focused on drugs that scored high on both human and mouse cell lines. Several such drugs are pan-kinase inhibitors (e.g. Staurosporine, FAK-inhibitor) with little target-specificity. For eEF2K, we identified 2 different inhibitors that scored very high on both mouse and human Pten/p53-deficient tumours. Given that eEF2K is downstream of mTOR, we decided to further interrogate this target.

Critique:
Page 13, line 2 (bottom), the authors probably meant " in many types of cancer"

Response:

Sentence corrected.

Critique:
Page 14, line 1, "Notably, we identified eEF2K inhibitors (?) under non-starving conditions." It is

unclear what this means.

Response:

It means that the cells were cultured under normal conditions (the cells were not starved). As we noted, Leprivier et al, 2013 showed that eEF2K inhibition suppressed growth of brain tumour cells under nutrient deprivation. In contrast, we found that eEF2K inhibition suppressed growth of TNBC cells even under nutrient rich conditions.

Critique:
Do these 6 lines indicate 4 mouse primary cultures and 2 human cell lines? The authors need to describe the results more clearly.

Response:

No. These are all human TNBC lines. We added “6 human” to clarify this issue

(“We therefore determined whether sensitivity of 6 human TNBC cell lines”)

Critique:
Page 16, the authors wrote "We estimated that the tumour suppressors... lost...in over 18% of these tumours." Is this statement based on any references or any data presented? The authors need to clarify.

Response:

We changed the word “estimated” with “found”. We now show the exact % of Pten/p53 low tumours in each BC subtype in **revised Figure 6B**.

Critique:
Page 16, line 8, the authors probably meant "Indeed, the inhibitors of these targets...."

Response:

Thanks. We have corrected this sentence.

2nd Editorial Decision 16 September 2014

Thank you for the submission of your revised manuscript to EMBO Molecular Medicine. We have now received the enclosed report from the referee who was asked to re-assess it. As you will see the reviewer is now globally supportive and I am pleased to inform you that we will be able to accept your manuscript pending final editorial amendments:

Please submit your revised manuscript within two weeks. I look forward to seeing a revised form of your manuscript as soon as possible.

***** Reviewer's comments *****

Referee #2 (Remarks):

Overall the authors have addressed a sufficient umber of my criticisms regarding presentation of the study, and the revised version is certainly improved. Note typos on p.12 top.
