# Supplementary material for: Combined deletion of Pten and p53 in mammary epithelium accelerates triple-negative breast cancer with dependency on eEF2K
Source: EMBO Mol Med. 2014 Oct 20;6(12):1542–60. doi: 10.15252/emmm.201404402 (PMC4287974; doi:10.15252/emmm.201404402)
Supplement: Supplementary file 2 — Supplementary Table S1 [file emmm0006-1542-sd2.pdf]

Combined Deletion of Pten and p53 in Mammary Epithelium Accelerates Triple-Negative Breast Cancer with Dependency on eEF2K

Jeff C. Liu[1], Veronique Voisin[2], Sharon Wang[1,3], Dong-Yu Wang[4,5], Robert A. Jones[1], Alessandro Datti[6,7], David Uehling[8], Rima Al-awar[8], Sean E. Egan[9,10], Gary D. Bader[2,10], Ming Tsao[4,11], Tak W. Mak[5,6,11], Eldad Zacksenhaus[1,3,11*]

SUPPLEMENTAL DATA

I. DETAILED MATERIALS AND METHODS

II. LEGENDS TO SUPPLEMENTAL FIGURES

I. DETAILED MATERIALS AND METHODS

PCR genotyping

Deletion of the Pten$^{f/f}$ allele was detected by PCR using the following primers: Forward: 5'GTCACCAGGATGCTTCTGAC3', Reverse: 5'ACTATTGAACAGAATCAACCC3'where Pten$^{f/f}$ results in a 335 bp product and Pten$^{\Delta f/f}$ in a 849 bp product; for p53 detection: Forward: 5'CACAAAAACAGGTTAAACCCAG3', Reverse: 5'AGCACATAGGAGGCAGAGAC3', where p53$^{f/f}$ gives a 288 bp product and p53$^{\Delta f/f}$ a 370 bp product.

Enrichment of lin$^-$ epithelial cells

Mammary tumors were minced with sterile razor blade, washed in PBS, digested in 100 U/ml collagenase/hyaluronidase (StemCell Technology, #07912) for 1 hour at 37ºC with occasional mixing, and washed once with 5X HBSS (Sigma, phenol red free, #H4891) + 2% FBS & 1 mM EDTA (HFE). Cell suspensions/digests were centrifuged at 1000 rpm, supernatant discarded, and pellet resuspended in 10 ml HBSS + 2% FBS & 1 mM EDTA (HFE) followed by passing through a 40 μm cell strainer (BD Falcon, #352340). Selective depletion of endothelial (anti-CD31, BD PharMingen) and hematopoietic cells (anti-CD45 and anti-TER119, StemCell Technologies) was accomplished with

magnetic beads using a Mammary Stem Cell Enrichment kit from StemCell Technology (#19757) at 4°C.

Mammosphere/Tumorsphere culturing *in vitro.* Cell suspension of lin$^-$ mammary epithelial cells or sorted cells from tumors or mammary glands were plated onto ultra low attachment plates (Corning, Costar #3471) in DMEM/F-12 HAM medium (Sigma #D8900) containing 20 ng/ml bFGF (Sigma # F0291), 20 ng/ml EGF (Sigma #E9644), 4 µg/ml of Heparin (Sigma #H4784) and B-27 supplement (1:50 dilution, GIBCO, #17504-044), and cultured at 37°C; 5% CO_2. Spheres were mechanically and enzymatically dissociated every 2-3 weeks in 1X (0.25%) Trypsin-EDTA solution (GIBCO #25200) for 1 min at RT, followed by passing through 25G needles.

Flow Cytometry Analysis and Sorting

For flow cytometry, we used anti-CD49f conjugated with R-phycoerythrin (CD49f-PE, clone GoH3, 5 µl/million cells, BD Pharmingen #555736), anti-CD24 conjugated with fluorescein isothiocyanate (CD24-FITC, clone M1/69, 0.25 µg/million cells, BD Pharmingen #553261), anti-Sca1 conjugated with R-phycoerythrin (Sca1-PE, clone E13-161.7, 0.25 µg/million cells, BD Pharmingen #553108). Cells were suspended in HBSS + 2% FBS & 1 mM EDTA (HFE) at 5 million cells/ml and incubated with indicated antibodies and cell-viability markers on ice for 30 minutes. After 3x washes in HFE, cells were re-suspended in HFE at 5 million cells/ml and kept on ice pending analysis. Single (fixed FSC-A/FSC-W ratio) and live cells (PI- or 7AAD-negative) were gated for analysis and sorting. For flow cytometry analysis, 7AAD (BD Pharmigen, Cat # 51-68981E) was used as the viability marker with FACS Calibur (Becton Dickinson, San Jose, CA). For sorting, Propidium Iodide (PI; BD

Pharmigen, Cat # 550825) was used for selecting live cells in 13 color FACS Aria(Becton Dickinson, San Jose, CA) with 488 nm Blue laser at 20 PSI, HSC-UHN Flow Cytometry Facility (Toronto).

Histology, TUNEL, Immuno-histochemistry and -fluorescence Staining

In vitro differentiation and immunocyto staining were performed as described previously (Liu et al, 2007) using rabbit anti-smooth muscle actin (SMA, 1:200 dilution, Novus Biologics, #600-531), mouse anti-keratin 18 (K18, 1:200 dilution, Fitzgerald, #RDI-PR061028), and rabbit anti-keratin 14 (K14, 1:200 dilution, Spring Bioscience, #E2624). Cells were washed in PBS (3X, 3min each) and incubated for 45 min with goat anti rabbit Alexa 488 (green, 1:200 dilution, Molecular probe, #A11008) or goat anti mouse Alexa 568 (red, 1:200 dilution, Molecular probe, #A11004). Nuclei were visualized with 4',6'-diamidino-2-phenylindole (DAPI, Sigma, #D9542). Slides were washed three times with PBS, mounted (DakoCytomation #S3023) and analyzed under Zeiss Axioskop 2 fluorescent microscope. Antibodies for IHC: keratin18 (K18, 1:50 dilution, Fitzgerald, #RDI-PR061028), rabbit keratin14 (K14, 1:200 dilution, Spring Bioscience, #E2624), mouse keratin 14 (K14, 1:100, Abcam, #ab7800), N-Cadherin (Novus Biologicals, 1:400 dilution, #NB200-592), Vimentin (SantaCruz, 1:50 dilution, #SC32322), keratin 5 (Covance, 1:200 dilution, #PRB-160P), keratin 6 (K6, Covance, 1:1000 dilution, #PRB-169B), smooth muscle actin (SMA, Novus Bilogicals, 1:200 dilution, #NB600-531), Desmin (Dako, 1;100, #M0760), estrogen receptor α (ER, Santa Cruz, 1:50 dilution, #SC542), cyclin D2 (Santa Cruz, 1:200 dilution, #SC593), Ki67 (Biocare Medical, 1:200, #CRM325, clone SP6), p53 (Santa Cruz, 1:200 dilution, #SC6243), and total β-catenin (BD Transduction, 1:200 dilution, #610154). TUNEL analysis was performed as previously described (Jiang et al, 2010).

Transplantation

Sorted cells, cells enzymatically isolated from tumorspheres, and 3 mm^3 tumor pieces from WAP-Cre:$Pten^{f/f}$, MMTV-Cre:$Pten^{f/f}$ or MMTV-Cre:$Pten^{f/f}$:$p53^{f/f}$ tumors were transplanted into #4 mammary glands of 3-5 week old immune-deficient female mice. Indicated number of cells were resuspended in 10 µl of media and mixed at 1:1 ratio with 10 µl matrigel (BD Bioscience #356234) on ice. The samples (total 20µl) were then injected into #4 mammary glands of female mice under isoflurane anesthesia. Mice were monitored for tumor formation for up to six months.

Western Analysis

Mammary tumour tissues or cultured cells were lysed with ice-cold RIPA buffer (PBS, 1% NP 40, 0.5 % (w/v) sodium deoxycholate, 0.1 % (w/v) sodium dodecyl sulfate (SDS), 1mM phenylmethylsulfonyl fluoride (PMSF), 60 µg/ml aprotinin, 5 mM DTT) in a Dounce homogenizer and immunoblotted as described (Liu et al, 2012). Blots were then incubated at 4°C overnight with antibodies against p-AKT (Ser473, 1:1000, Cell Signaling Technology), anti-phospho-eEF2 (T56, Cell Signaling, Cat# 2331). Tubulin (1:3000, Cell Signaling Technology), LC3b (1:1000, Cell Signaling Technology, #2775) diluted with 1% BSA in PBS, followed by anti-rabbit IgG HRP antibodies (1:3000, Cell Signaling) for 1 hour at room temperature. Chemiluminescence was detected using Super Signal West Dura (Pierce); images captured with BioRad Flour-S-Max Multimager, equipped with a Nikon CCD camera.

Bioinformatics. Microarray analysis with mouse tumor models was carried out using Affymetrix Mouse Gene 1.0 ST with 500 ng of total RNA isolated by double Trizol extractions (Centre for

Applied Genomics, Hospital for Sick Children, Toronto). Microarray data were normalized using RMA method via Partek software and log2-transformed gene expression values were obtained.

To compare pathway activities in mouse tumors with human breast samples we calculated values of pathway activities as described (Gatza et al, 2010) for samples from cohorts GSEs 1456, 1561, 2034, 3744, 4922, 5460, 5764, 6532, and 6596. We also calculated pathway activities for four more cohorts (GSEs 2603, 5327, 11121, and 25066) and mouse models using the same method (Table S1N-O). Metastasis-Free Survival (MFS) information is annotated for cohorts GSEs 2034, 2603, 5327, 6532, 11121, and 25066. The intrinsic subtypes of BCs were classified using PAM50 (Table S1P). As PAM50 does not distinguish basal-like from claudin-low subtypes, we identified claudin-low patients from PAM50-defined basal (TN) samples using the claudin-low signature (Table S1B) (Prat et al, 2010). Data from above GSEs were integrated with GSE18229, which includes both pre-identified claudin-low and basal-like tumors, using "Distance Weighted Discrimination" (DWD). In addition, unsupervised hierarchical clustering (complete linkage) was used to identify additional claudin-low tumors (Figure S6A). For human p53 activity, we used median value of p53 mutants in GSE4922 (0.15, Figure 5A) for normalization/centering. Values of pathway activities were median-centered and visualized as heatmaps. Pearson's correlation was performed to determine potential relations in pathway activities in different mouse models and human BC subtypes. Pten gene expression was determined using the least variable probeset (204054_at) shared by all platforms (GPL96 and GPL570). To compare BC subtypes with our mouse models, median-centered values of mouse microarray data were integrated with GSE3165 by DWD using shared intrinsic genes ((Herschkowitz et al, 2007); Table S1A) and the claudin-low signature ((Prat et al, 2010); Table S1B). For side-by-side comparison of mouse tumors with human claudin-low and basal subtypes in GSE18229, several groups of genes (EMT, Basal-B and Hypoxia **Table S1C-E**) were median-centered and visualized by

heatmaps. miRNAs related to EMT and expression of miRNA processing proteins Trp63 and Dicer1 were also visualized by heatmaps using median-centered values (Table S1F).

Generation and assessment of prognostic signature for claudin-low breast cancer (WCLS)

Microarray data were normalized using RMA method via Partek software and log2 transformed gene expression values were obtained. ANOVA analysis with FDR correction was performed between WAP-Cre:Pten$^{f/f}$:p53$^{f/f}$ and MMTV-Cre:Pten$^{f/f}$:p53$^{f/f}$ tumors to identify significantly (FDR q-value<0.05) and differentially (>2.0 fold) expressed genes. Prognostic value of these genes was assessed using 96 Claudin-low patients (**Figure 3D**) from six GPL96 cohorts (GSEs 2034, 2603, 5327, 6532, 11121, and 25066) containing Metastasis-Free Survival (MFS) Data. Each dataset was analyzed independently by obtaining RMA normalized expression value of the individual cohort for log2 transformation and median-centering. Score for Signature Match (SSM) (Liu et al, 2012) was used to differentiate the samples, and SSM>0 was considered to be a match to the signature. Kaplan-Meier and Survival analysis were performed with PAST program (P.D. Ryan and Ø. Hammer, University of Oslo) and p-value was calculated by Wilcoxon method. Hazard ratios were obtained using the COX Proportional Hazards Survival Regression method. Heatmaps and dendrograms were generated by JAVA tree-view.

Gene set enrichment analysis

Gene expression data were analyzed using GSEA (Subramanian et al, 2005) with parameters set to 2000 gene-set permutations and gene-sets size between 8 and 500. Genes were ordered using the logFC corresponding to each pair-wise comparison. The gene-sets included in the GSEA analyses were obtained from KEGG, MsigDB-c2, NCI, Biocarta, IOB, Netpath, Human Cyc, Reactome and the Gene

Ontology (GO) databases, updated March 2012 (http://baderlab.org/GeneSets). An enrichment map(version 1.2 of Enrichment Map software (Merico et al, 2011) was generated for each comparison using enriched gene-sets with a nominal p-value <0.05 and the overlap coefficient set to 0.5.

Senescence β-Galactosidase Staining

Cells were plated on collagen coated cover slips (BD Biocoat; VWR #354089) and cultured in DMEM/F-12 HAM medium (Sigma #D8900) containing 20 ng/ml bFGF (Sigma # F0291), 20 ng/ml EGF (Sigma #E9644), 4 µg/ml of Heparin (Sigma #H4784) and B-27 supplement (1:50 dilution, GIBCO, #17504-044) at 37°C with 5% CO_2 for 3 days. The cells were then fixed with 4% paraformaldehyde for 5 minutes at 25 °C and washed 3 times with PBS. The Senescence β-Gal staining solution (30 mM Citric Acid/Phosphate Buffer, pH 6.0 with 5 mM $K_3Fe(CN)_6$, 5 mM$K_4Fe(CN)_6$, 2 mM $MgCl_2$, 150 mM NaCl, and 1 mg/ml X-Gal) was added and the cells were stained at 37°C for 12 hours. Cover slips were washed in PBS and mounted in 30% glycerol (in PBS).

Kinase Inhibitor Screening, IC50 and MTT assay

238 compounds targeting 154 different kinases were screened using robotic Biomek FX liquid handler equipped with a pintool for automated compound dispensing. Assays were carried out in a 384-well format, using 300 cells/well. Compounds, resuspended in DMSO as 1mM stock solutions, were added in a volume of 200 ml to 3µM final concentration. Cell viability was monitored by incubation with Alamar Blue (Life Technologies, #DAL1025) at 10x dilution for 4 hours, and read by FLUOstar Optima reader (BMG Labtech) at 590nm emission wavelength with 530-560nm excitation wavelength. As a reference for 100% activity, each plate included 32 wells with cells treated with vehicle only, and background was measured with media in the absence of cells. Assays were optimized for dynamic

range (100% activity/background, >10), while variability was consistently found to be low (i.e. CV <10%). For validation/IC50/further analyses, cells were plated at 500 cells/well concentration in 100 µl media into 96-well plates and treated the next day with indicated compounds at increasing concentrations (1, 3, 10, 30, 100 µM) for 3 days. Cell viability was measured by adding 3-(4,5-dimethylthiazol-2-yl)-2,5-diphenyl-tetrazolium bromide (MTT) to a final concentration of 1 mg/ml for 2 h at 37°C, followed by PBS wash and re-suspension in 100 µl DMSO overnight. Colorimetric reading at 570 nm was performed using a microplate reader (Molecular Devices).

Xenograft assays

Pten:p53-mutant mouse (200,000 cells/injection) or human BT549 (1 million cells/injection) tumor cells were resuspended in 20 µl of media/matrigel mixture (1:1) and injected into #4 mammary glands of NOD/SCID females (9 mice per group). NH125 (1 mg/kg, dissolved in PBS with 2% DMSO) and SP600125 (60 mg/kg and 30 mg/kg, dissolved in DMSO) were administered intraperitoneally as described (Figure 8A legend). Control mice were injected with vehicle alone at the same weight/volume ratio.

Additional Statistical Analysis

Parametric paired samples were analyzed by student t-test; non-parametric paired samples by Mann-Whitney test. Significance of comparing multiple samples was calculated using ANOVA and the Tukey test for *post hoc* analysis. Chi-square test was used to compare two categorical variables. For multi-categorical variable comparisons, Kruskal-Wallis test was performed. Kaplan-Meier and Survival analysis were performed using the PAST program (P.D. Ryan and Ø. Hammer, University of Oslo) and p-value was calculated using the Wilcoxon method. Differences between values were

considered statistically significant at $p < 0.05$. TIC frequency and 95% confidence intervals were calculated using the L-Calc software from www.stemcell.com. For meta-analysis, TNBC cell lines (BT549, HCC38, HCC1937, MDAMB157, MDAMB436, & MDAMB468) with varying Akt activities were treated at 6 different concentrations (μM) of TX-1918 (0.03, 0.1, 0.3, 1.0, 3.0, & 10.0) or doxorubicin (0.01, 0.03, 0.1, 0.3, 1.0, & 3.0) in duplicates for 3 days and cell viability was determined by MTT assay. IC_{50} was calculated using package "ic50" in R. 7 experiments were completed for TX-1918 and 5 for doxorubicin. Correlation coefficient of Akt activity with IC_{50} values was calculated for each experiment and subjected to meta-analysis using package "metaphor" in R. Meta-analysis was performed using random effects model with DerSimonian-Laird for tau^2 estimator on heterogeneity.

II. LEGENDS TO SUPPLEMENTAL FIGURES

Figure S1. Epithelial marker analysis of WAP-Cre:Pten$^{f/f}$ and MMTV-Cre:Pten$^{f/f}$ mammary tumors

(A) Expression of cytokeratin6 (K6), K14, vimentin, K5, estrogen receptor α (ER), β-catenin (β-Cat) and Cyclin D2 (D2) in WAP-Cre:Pten$^{f/f}$ and MMTV-Cre:Pten$^{f/f}$ tumors.

(B) Nuclear co-expression of total β-catenin (β-Cat) and Cyclin D2 (D2) by confocal microscopy in WAP-Cre:Pten$^{f/f}$ versus MMTV-Wnt1 tumors. Note nuclear localization of β-catenin in cells expressing Cyclin D2 in Pten-deficient tumor cells.

Figure S2. EMT marker analysis of WAP-Cre:Pten$^{f/f}$ and MMTV-Cre:Pten$^{f/f}$ mammary tumors

(A) Representative staining for cytokeratin14 (K14), K18, vimentin, K5, smooth muscle actin (SMA), K6, and estrogen receptor α (ERα) in MMTV-Cre:Ptenf/f:p53f/f versus MMTV-Cre:p53f/f tumors.

(B) Expression of the EMT marker Desmin in indicated tumors. MMTV-Neu and MMTV-Wnt1 tumors served as negative controls.

Figure S3. Analysis of WAP-Cre:Pten$^{f/f}$ and MMTV-Cre:Pten$^{f/f}$ models and human TNBCs using the basal B signature, microRNAs, Trp63, and Dicer1

(A) Using the Basal B signature, MMTV-Neu, MMTV-Cre:p53$^{f/f}$, WAP-Cre:Pten$^{f/f}$, MMTV-Cre:Pten$^{f/f}$, WAP-Cre:Pten$^{f/f}$:p53$^{f/f}$ and MMTV-Cre:Pten$^{f/f}$:p53$^{f/f}$ mouse tumors were compared with human Claudin-low and basal-like BC samples from GSE3165.

(B) Pten/p53-deficient tumors have significantly lower expression of miR200a ($p = 0.0024$), mir200b ($p = 0.000173$), miR200c ($p = 0.0113$), miR429 ($p = 0.000984$) and miR205 ($p = 9.87 \times 10^{-5}$) than Pten-deficient tumors; ANOVA with Tukey test for post hoc.

(C) Expression of indicated microRNAs involved in EMT in MMTV-Neu, MMTV-Cre:p53$^{f/f}$, WAP-Cre:Pten$^{f/f}$, MMTV-Cre:Pten$^{f/f}$, WAP-Cre:Pten$^{f/f}$:p53$^{f/f}$ and MMTV-Cre:Pten$^{f/f}$:p53$^{f/f}$ tumors.

(D) Expression of Trp63 and Dicer1 in MMTV-Neu, MMTV-Cre:p53$^{f/f}$, WAP-Cre:Pten$^{f/f}$, MMTV-Cre:Pten$^{f/f}$, WAP-Cre:Pten$^{f/f}$:p53$^{f/f}$ and MMTV-Cre:Pten$^{f/f}$:p53$^{f/f}$ tumors.

Figure S4. GSEA analysis of WAP-Cre:Pten$^{f/f}$:p53$^{f/f}$ and MMTV-Cre:Pten$^{f/f}$:p53$^{f/f}$ tumors and analysis of WCLS, BLBC and Taube/Mani EMT signatures

(A) Full GSEA analysis of WAP-Cre:Pten$^{f/f}$:p53$^{f/f}$ (red) versus MMTV-Cre:Pten$^{f/f}$:p53$^{f/f}$ (blue) tumors using canonical pathways (c2.all, v4.0, from Broad Institute). Green lines connect overlapping pathways. The size of the circle corresponds with the level of enrichment whereas the thickness of the lines corresponds with the degree of overlap. WCLS gene group is shown as a yellow triangle with related pathways connected to it through red lines.

(B) Kaplan-Meier Metastasis-Free Survival (% MFS) curve for HER2, Luminal A and Luminal B BC patients with WCLS, BLBC, and Taube/Mani EMT signatures. Note lack of significant prognostication of these tumor subtypes.

(C) Comparison of WCLS vs. BLBC on basal-like breast cancer. All 24 genes (7 up-regulated and 17 down-regulated) of WCLS and 14 genes of BLBC (9 up-regulated and 5 down-regulated) were present on Affymetrix U133A platform. Analysis was performed with 245 Basal-like

patients from 6 U133A cohorts (GSEs 2034, 2603, 5327, 6532, 11121, and 25066 with Metastasis-Free Survival (MFS) Data). 1000 random sets of signatures with the same number of genes were generated from atmosphere background noise (random.org) and SSM algorithm was employed to differentiate samples. The signatures were ranked by HR and compared with WCLS (red boxes) and BLBC (blue boxes). The % signatures with significant HR > 1.0 is also listed at the bottom. Comparing to 1000 sets of random signatures, BLBC ranked 2nd for Basal-like MFS samples while WCLS ranked at #141.

Figure S5. Pathway analysis comparing MMTV-Cre:Pten$^{f/f}$, MMTV-Cre:p53$^{f/f}$ and MMTV-Cre:Pten$^{f/f}$:p53$^{f/f}$ tumors

(A) Complete GSEA pathway analysis comparing Claudin-low MMTV-Cre:Pten$^{f/f}$:p53$^{f/f}$ (red) with MMTV-Cre:p53$^{f/f}$ (blue) tumors.

(B) GSEA pathway analysis comparing all MMTV-Cre:Pten$^{f/f}$:p53$^{f/f}$ (red) with MMTV-Cre:p53$^{f/f}$ (blue) tumors.

(C) GSEA pathway analysis comparing tumors from MMTV-Cre:Pten$^{f/f:p53f/f}$ (red) with MMTV-Cre:Pten$^{f/f}$ (blue) mice.

(D) GSEA pathway analysis comparing tumors from MMTV-Cre:p53$^{f/f}$ (red) with MMTV-Cre:Pten$^{f/f}$ (blue) mice.

(E) Representative images of Ki67 and TUNEL staining of MMTV-Neu, MMTV-Wnt1, WAP-Cre:Pten$^{f/f}$, MMTV-Cre:Pten$^{f/f}$, MMTV-Cre:p53$^{f/f}$ and MMTV-Cre:Pten$^{f/f}$:p53$^{f/f}$ tumors.

(F) Statistical analysis comparing percentage of Ki67 and TUNEL positive cells in MMTV-Cre:Pten$^{f/f}$:p53$^{f/f}$ tumors with other mouse models; ANOVA with Tukey test for post hoc. Significant differences are highlighted in red.

Figure S6. Identification of Claudin-low BC using Prat/Perou claudin-low signature

(A) Unsupervised hierarchical clustering analysis using the Prat/Perou claudin-low signature for TNBC samples from GSEs 1456, 1561, 2034, 2603, 3744, 4922, 5327, 5460, 5764, 6532, 6596, 11121 and 25066 (red boxes). Microarray data were integrated by DWD with GSE18229, which had been pre-assigned by Prat & Perou as claudin-low (green) or Basal-like (blue) BC samples.

(B) Top, immunoblot analysis of phospho-Akt (Ser473) in indicated tumors and wild-type mammary gland (WT). Tubulin served as loading control. Bottom, ratios of phospho-Akt/Tubulin.

(C) Induction of MYC pathway activity in Pten/p53-deficient mouse tumors (left) and human claudin-low BC (right). Values were normalized by median and average activity (Ave) with statistical significance (p-value from ANOVA with Tukey test for post hoc) calculated for $Pten^{f/f}$:$p53^{f/f}$ and claudin-low tumors (red boxes) vs. other BC subtypes.

Figure S7. Drug sensitivity and correlation with signaling pathways in Pten/p53-deficient tumors

(A) List of compounds ranked by levels of inhibition of 4 primary MMTV-Cre:$Pten^{f/f}$:$p53^{f/f}$ tumor cell lines and human BT549 and HCC1937 cell lines. Fold change and T-scores are indicated. PI3K/AKT pathway inhibitors are highlighted in red.

(B) BI78D3-mediated inhibition of primary MMTV-Cre:$Pten^{f/f}$:$p53^{f/f}$ tumor cells ($Pten^{\Delta f}$:$p53^{\Delta f}$) versus the HC11 mammary epithelial cell line.

(C) IC_{50} values for TX-1918 (n=7) and control doxorubicin (n=5) were determined in TNBC cell lines (HCC38, HCC1937, BT549, MDAMB157, MDAMB436, MDAMB468). Correlation

coefficient (r) of IC_{50} values with activities of all 18 pathways was calculated for each experiment by linear regression using meta-analysis. AKT pathway activity shows the most consistent and highest correlation with sensitivities of TNBC cells to eEF2K inhibition (TX-1918).

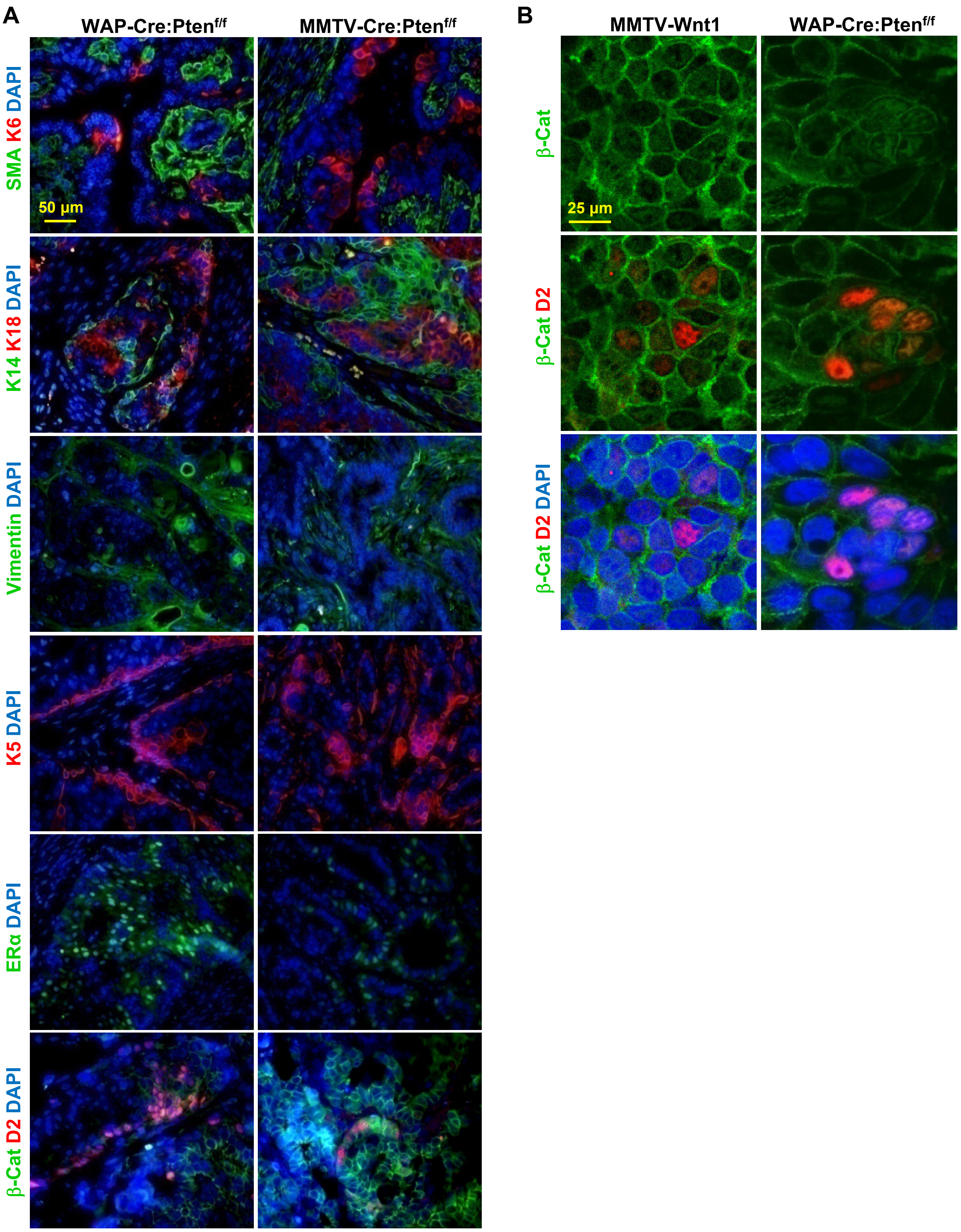
A
WAP-Cre:Pten$^{f/f}$
MMTV-Cre:Pten$^{f/f}$
SMA K6 DAPI
50 µm
K14 K18 DAPI
Vimentin DAPI
K5 DAPI
ERα DAPI
β-Cat D2 DAPI
B
MMTV-Wnt1
WAP-Cre:Pten$^{f/f}$
β-Cat
25 µm
β-Cat D2
β-Cat D2 DAPI

Figure S1

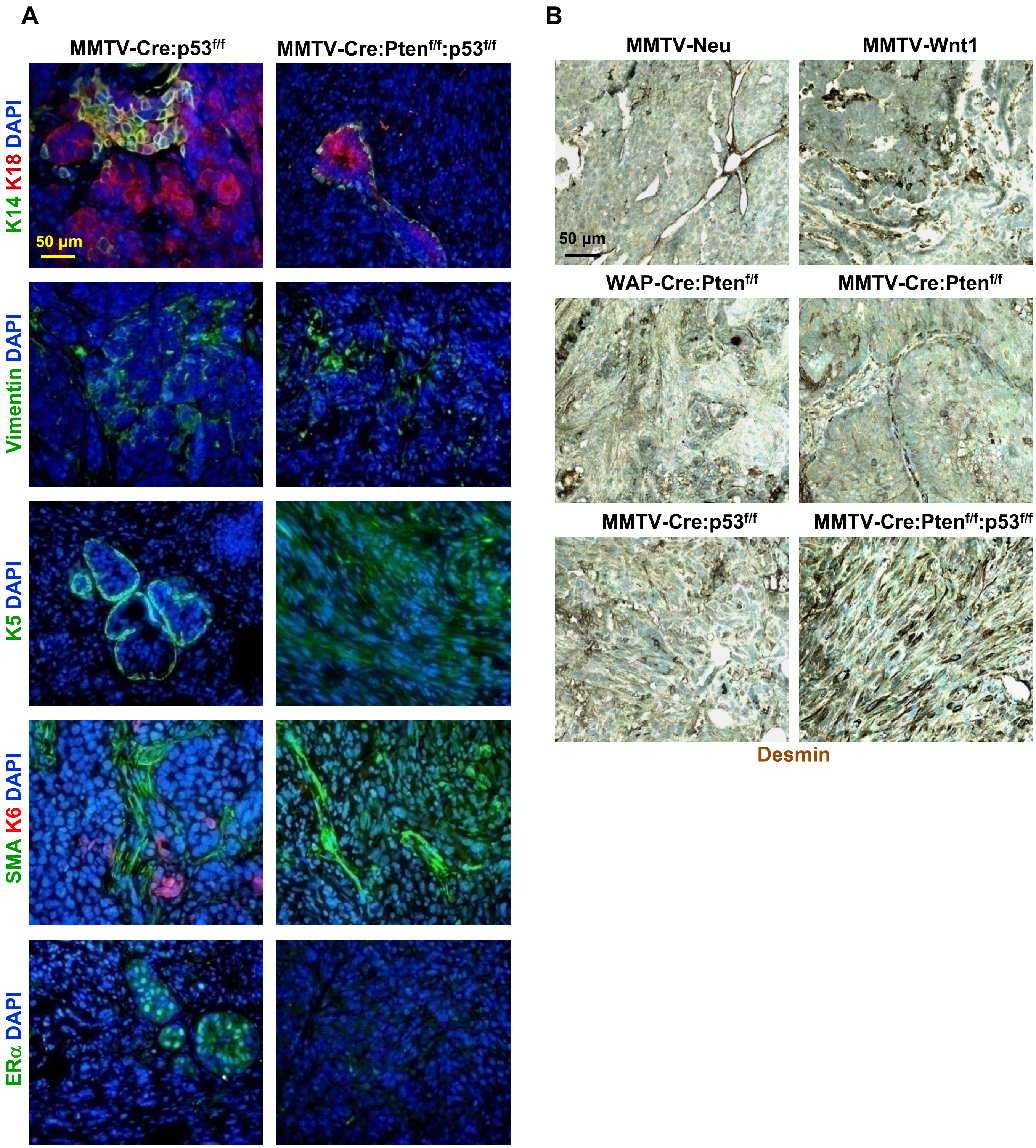
A
MMTV-Cre:p53f/f
MMTV-Cre:Ptenf/f:p53f/f
K14 K18 DAPI
50 µm
Vimentin DAPI
K5 DAPI
SMA K6 DAPI
ERα DAPI
B
MMTV-Neu
MMTV-Wnt1
50 µm
WAP-Cre:Ptenf/f
MMTV-Cre:Ptenf/f
MMTV-Cre:p53f/f
MMTV-Cre:Ptenf/f:p53f/f
Desmin

Figure S2

A

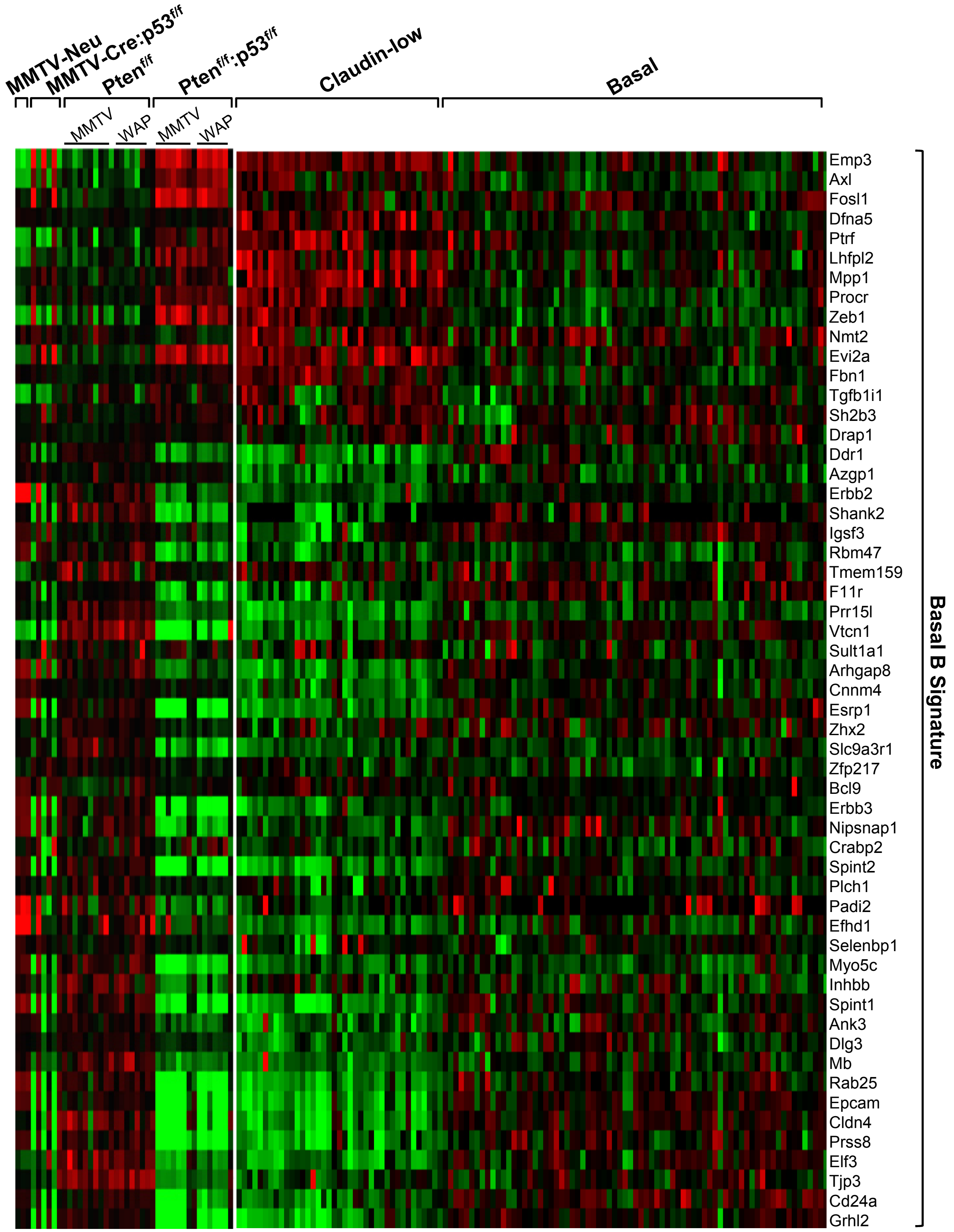
MMTV-Neu
MMTV-Cre:p53f/f
Ptenf/f
Ptenf/f:p53f/f
Claudin-low
Basal
MMTV
WAP
MMTV
WAP
Emp3
Axl
Fosl1
Dfna5
Ptrf
Lhfpl2
Mpp1
Procr
Zeb1
Nmt2
Evi2a
Fbn1
Tgfb1i1
Sh2b3
Drap1
Ddr1
Azgp1
Erbb2
Shank2
Igsf3
Rbm47
Tmem159
F11r
Prr15l
Vtcn1
Sult1a1
Arhgap8
Cnnm4
Esrp1
Zhx2
Slc9a3r1
Zfp217
Bcl9
Erbb3
Nipsnap1
Crabp2
Spint2
Plch1
Padi2
Efhd1
Selenbp1
Myo5c
Inhbb
Spint1
Ank3
Dlg3
Mb
Rab25
Epcam
Cldn4
Prss8
Elf3
Tjp3
Cd24a
Grhl2
Basal B Signature

Figure S3A

B

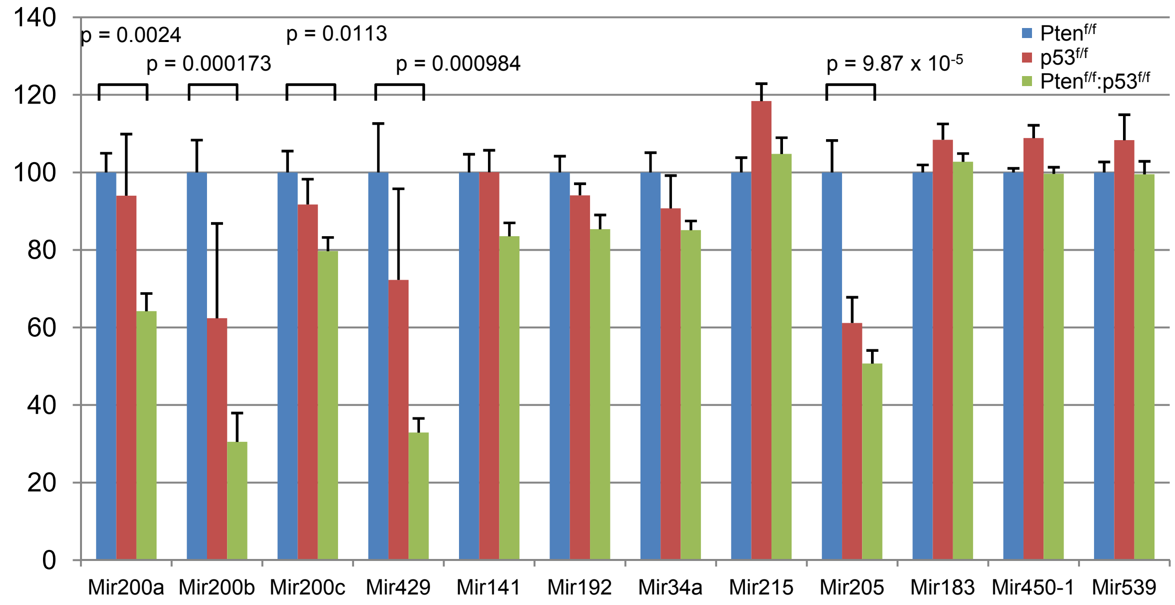

C

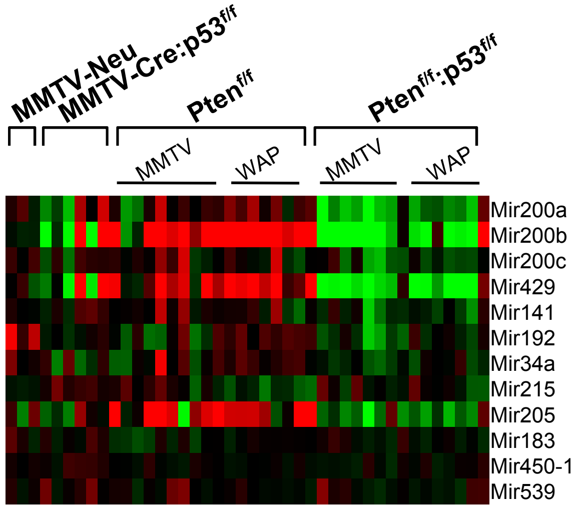

D

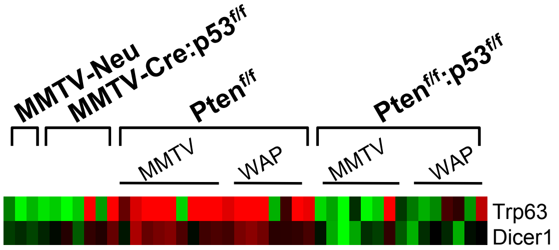

	Neu	p53f/f	Ptenf/f	Ptenf/f: p53f/f	
Ave	248	339	893	367	Trp63
	1122	1310	1634	1075	Dicer1
ANOVA	0.898	0.998	0.0199	--	Trp63
	0.98	0.23	0.000389	--	Dicer1

Figure S3B-D

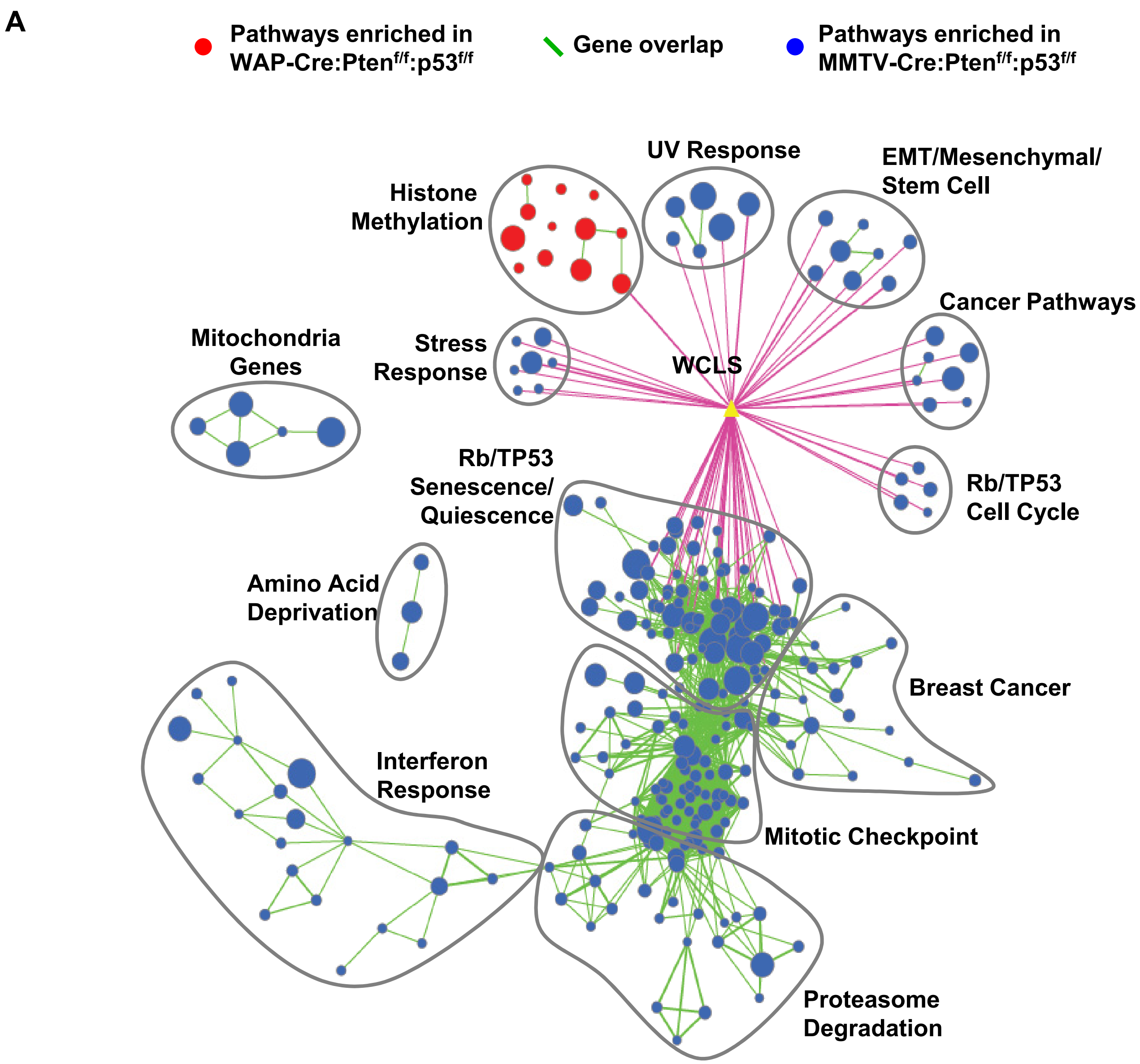
A
Pathways enriched in WAP-Cre:Pten$^{f/f}$:p53$^{f/f}$
Gene overlap
Pathways enriched in MMTV-Cre:Pten$^{f/f}$:p53$^{f/f}$
UV Response
EMT/Mesenchymal/ Stem Cell
Histone Methylation
Cancer Pathways
Mitochondria Genes
Stress Response
WCLS
Rb/TP53 Senescence/ Quiescence
Rb/TP53 Cell Cycle
Amino Acid Deprivation
Breast Cancer
Interferon Response
Mitotic Checkpoint
Proteasome Degradation

Figure S4A

B

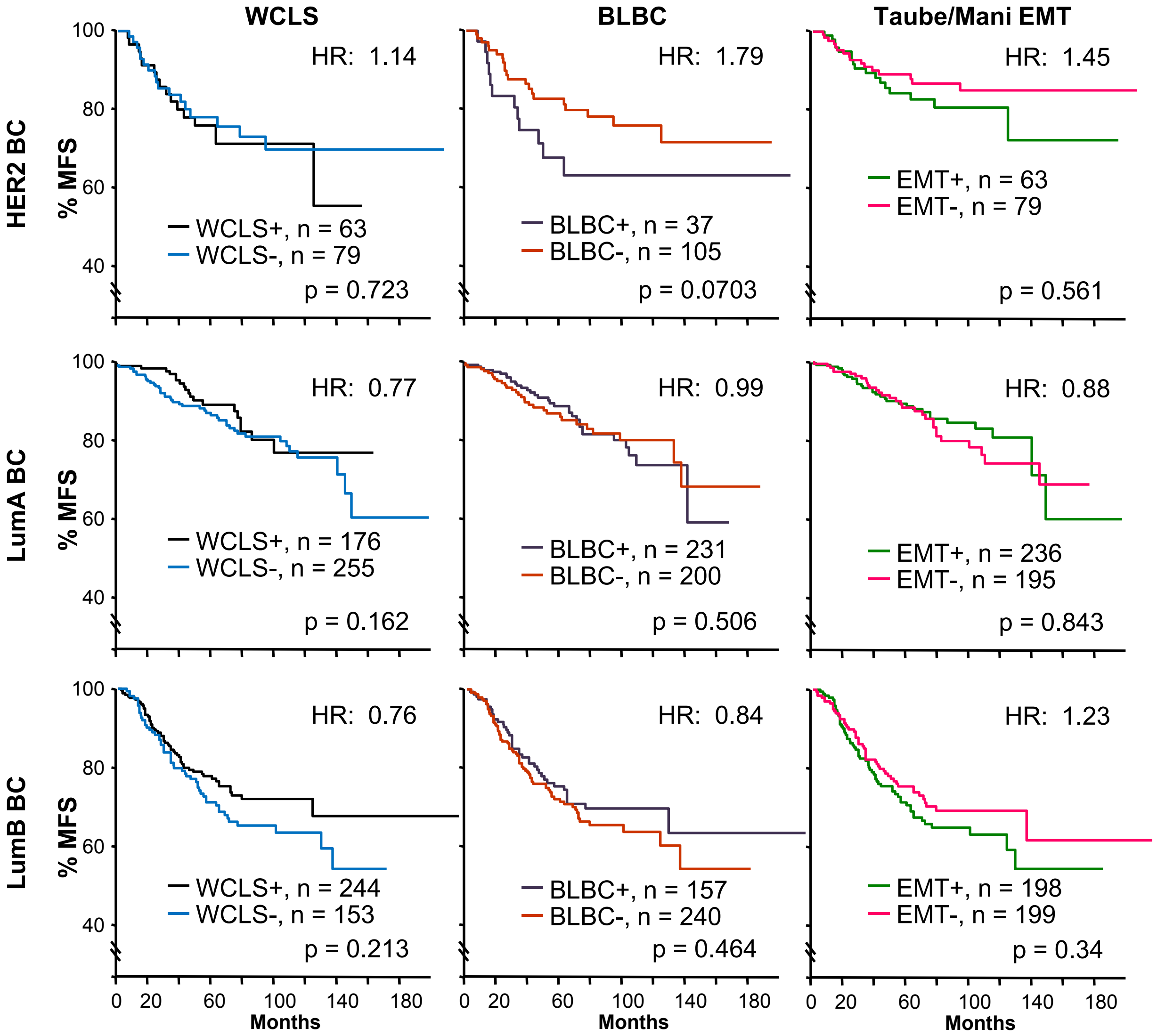

C

WCLS

Basal-Like Patients

MFS (n = 245)

Rank	HR:	p-value
1	2.72	0.00114
2	2.51	0.000845
3	2.49	0.00315
4	2.48	0.00145
5	2.45	0.00152
6	2.45	0.00141
7	2.44	0.00695
8	2.32	0.00871
9	2.28	0.0115
10	2.26	0.0165
11	2.25	0.00855
12	2.24	0.00331
13	2.22	0.0144
14	2.22	0.00627
15	2.21	0.0263
16	2.19	0.00593
17	2.15	0.00506
141	1.50	0.187

4.8% Random Signatures are Significant (p<0.05) **3.9%** have HR>1; **WCLS** ranked #141.

BLBC

Basal-Like Patients

MFS (n = 245)

Rank	HR:	p-value
1	2.40	0.00177
2	2.13	0.0121
3	2.11	0.0309
4	2.07	0.0209
5	2.03	0.0144
6	1.94	0.0379
7	1.92	0.0176
8	1.92	0.0509
9	1.91	0.018
10	1.91	0.0182
11	1.90	0.0307
12	1.87	0.0231
13	1.85	0.0716
14	1.78	0.0508
15	1.76	0.0418
16	1.72	0.0665
17	1.72	0.053
18	1.71	0.0582

5.0% Random Signatures are Significant (p<0.05) **1.2%** have HR>1; **BLBC** ranked #2.

Figure S4B-C

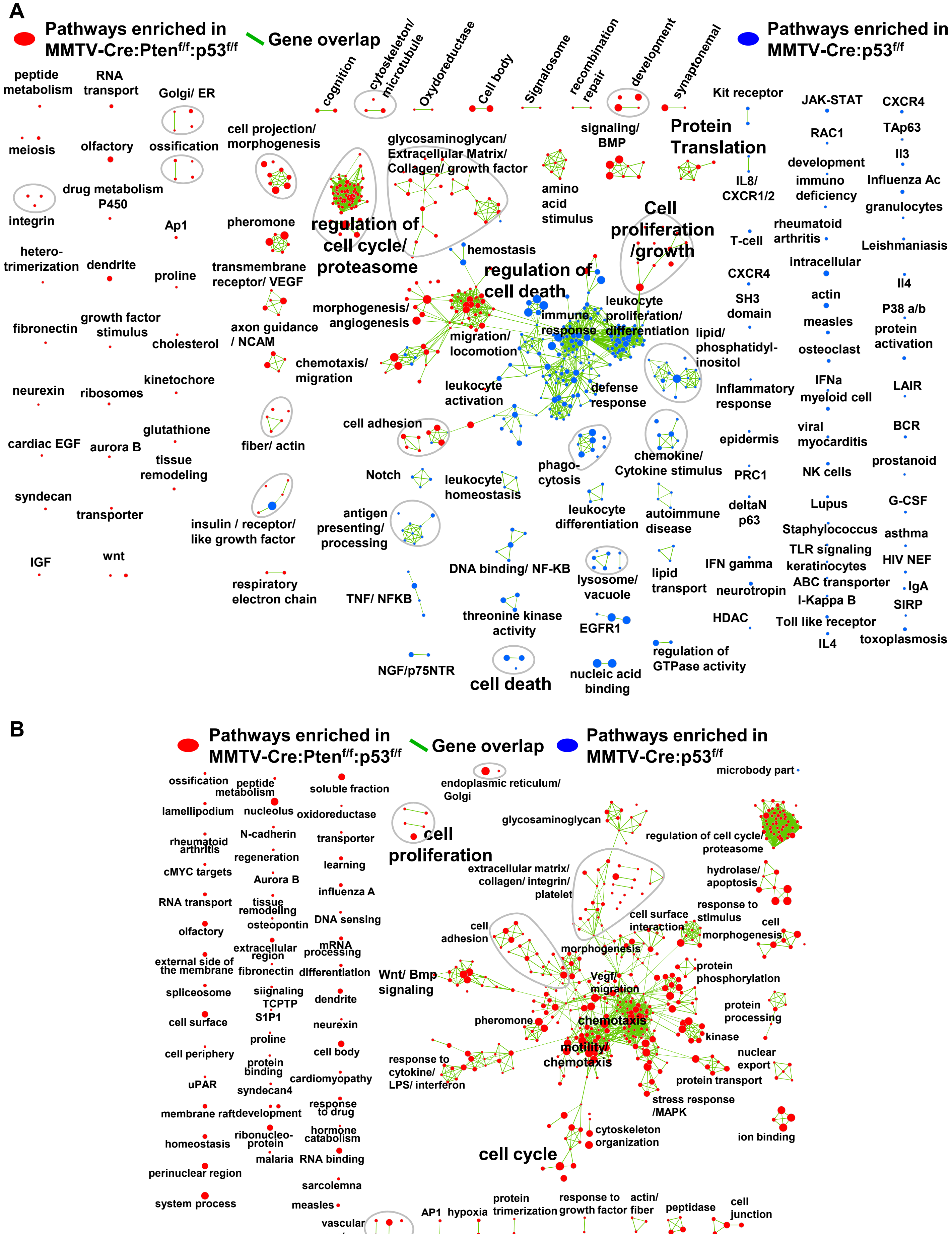
A
Pathways enriched in MMTV-Cre:Pten$^{f/f}$:p53$^{f/f}$
Gene overlap
Pathways enriched in MMTV-Cre:p53$^{f/f}$
peptide metabolism
RNA transport
Golgi/ ER
cognition
cytoskeleton/ microtubule
Oxydoreductase
Cell body
Signalosome
recombination repair
development
synaptonemal
Kit receptor
JAK-STAT
CXCR4
meiosis
olfactory
ossification
cell projection/ morphogenesis
signaling/ BMP
Protein Translation
RAC1
TAp63
glycosaminoglycan/ Extracellular Matrix/ Collagen/ growth factor
Il3
development
integrin
drug metabolism P450
amino acid stimulus
IL8/ CXCR1/2
immuno deficiency
Influenza Ac
granulocytes
Ap1
pheromone
regulation of cell cycle/ proteasome
Cell proliferation /growth
rheumatoid arthritis
T-cell
hetero-trimerization
dendrite
hemostasis
Leishmaniasis
proline
transmembrane receptor/ VEGF
regulation of cell death
intracellular
CXCR4
Il4
fibronectin
growth factor stimulus
morphogenesis/ angiogenesis
leukocyte proliferation/ differentiation
SH3 domain
actin
P38 a/b
axon guidance / NCAM
immune response
measles
protein activation
cholesterol
migration/ locomotion
lipid/ phosphatidyl-inositol
osteoclast
chemotaxis/ migration
kinetochore
IFNa
neurexin
ribosomes
leukocyte activation
defense response
Inflammatory response
myeloid cell
LAIR
glutathione
cell adhesion
viral myocarditis
BCR
cardiac EGF
aurora B
fiber/ actin
epidermis
tissue remodeling
chemokine/ Cytokine stimulus
prostanoid
phago-cytosis
Notch
leukocyte homeostasis
PRC1
NK cells
syndecan
leukocyte differentiation
autoimmune disease
deltaN p63
Lupus
G-CSF
transporter
insulin / receptor/ like growth factor
antigen presenting/ processing
Staphylococcus
asthma
TLR signaling
IGF
wnt
DNA binding/ NF-KB
lysosome/ vacuole
lipid transport
IFN gamma
keratinocytes
HIV NEF
respiratory electron chain
ABC transporter
IgA
TNF/ NFKB
neurotropin
I-Kappa B
SIRP
threonine kinase activity
HDAC
Toll like receptor
EGFR1
IL4
toxoplasmosis
regulation of GTPase activity
NGF/p75NTR
cell death
nucleic acid binding
B
Pathways enriched in MMTV-Cre:Pten$^{f/f}$:p53$^{f/f}$
Gene overlap
Pathways enriched in MMTV-Cre:p53$^{f/f}$
microbody part
ossification
peptide metabolism
soluble fraction
endoplasmic reticulum/ Golgi
lamellipodium
nucleolus
oxidoreductase
glycosaminoglycan
rheumatoid arthritis
N-cadherin
transporter
cell proliferation
regulation of cell cycle/ proteasome
regeneration
cMYC targets
learning
extracellular matrix/ collagen/ integrin/ platelet
hydrolase/ apoptosis
Aurora B
influenza A
RNA transport
tissue remodeling
response to stimulus
osteopontin
DNA sensing
cell surface interaction
cell morphogenesis
olfactory
cell adhesion
extracellular region
mRNA processing
morphogenesis
external side of the membrane
fibronectin
differentiation
protein phosphorylation
Wnt/ Bmp signaling
Vegf/ migration
spliceosome
siignaling TCPTP
dendrite
S1P1
protein processing
cell surface
neurexin
pheromone
chemotaxis
kinase
proline
cell periphery
cell body
motility/ chemotaxis
nuclear export
protein binding
response to cytokine/ LPS/ interferon
uPAR
cardiomyopathy
protein transport
syndecan4
stress response /MAPK
response to drug
membrane raft
development
hormone catabolism
cytoskeleton organization
ion binding
homeostasis
ribonucleo-protein
malaria
RNA binding
cell cycle
perinuclear region
sarcolemna
system process
measles
protein trimerization
response to growth factor
actin/ fiber
peptidase
cell junction
AP1
hypoxia
vascular system

Figure S5A-B

C

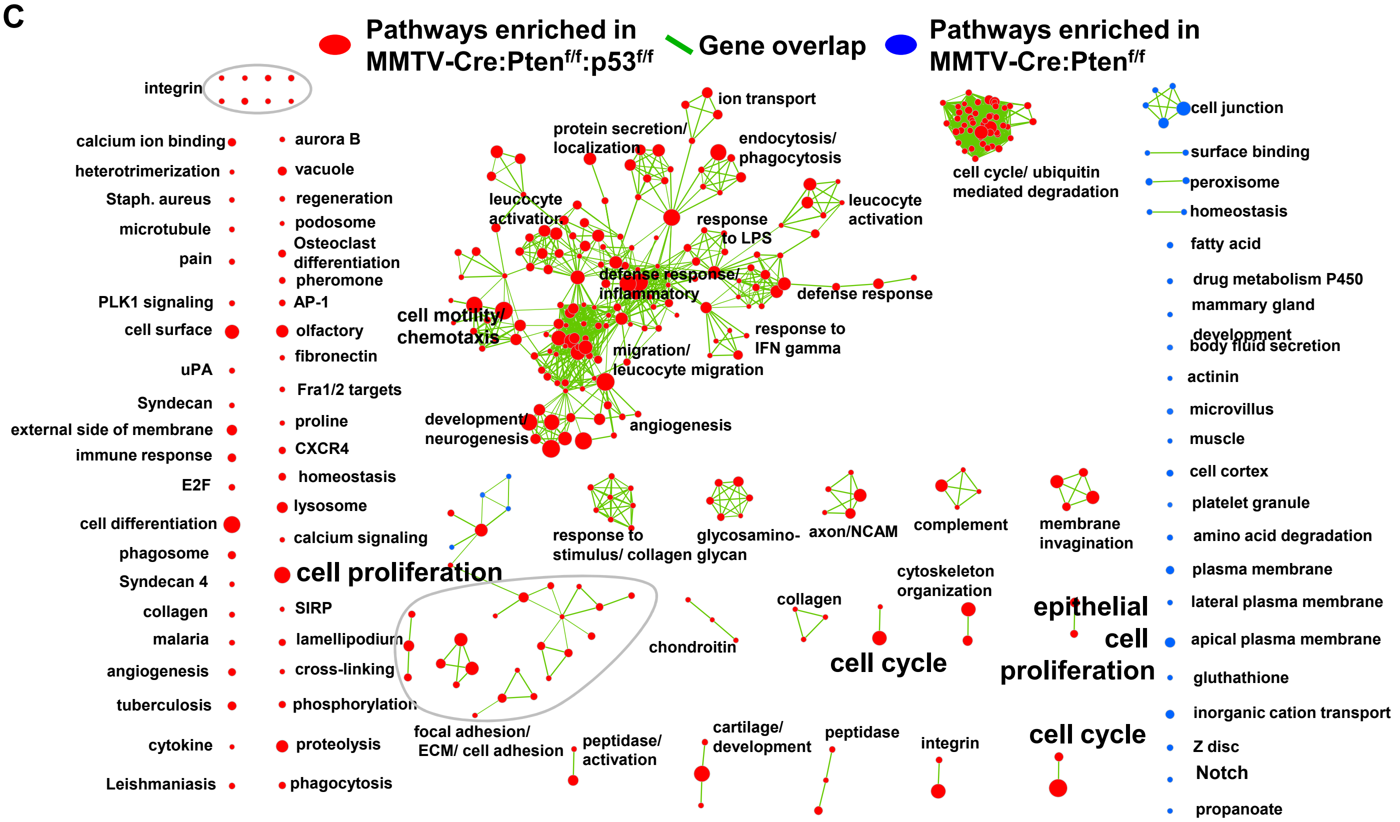

D

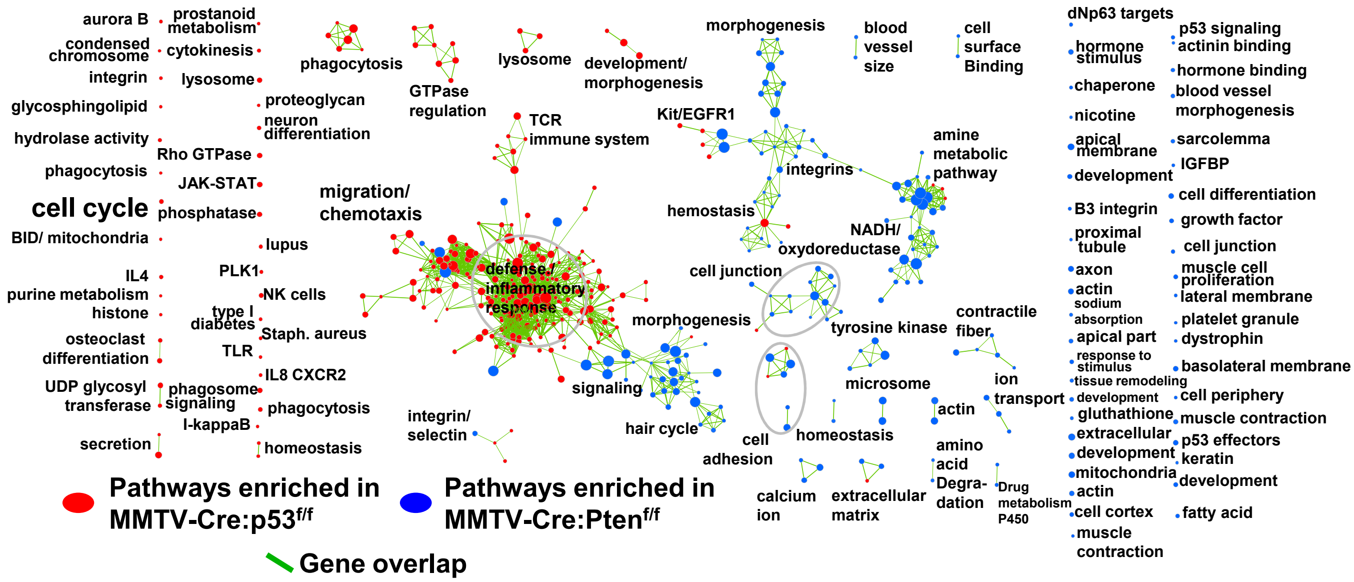

E

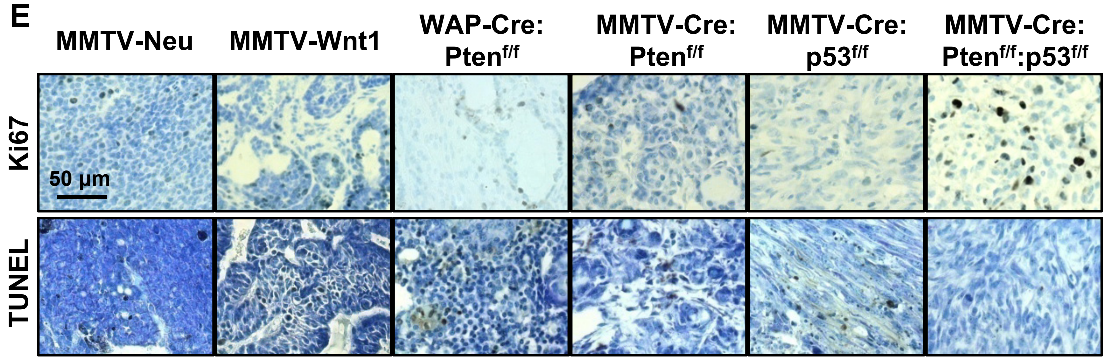

F

Ki67

Type	Pten$^{f/f}$:p53$^{f/f}$
Neu	0.00199
Wnt1	0.00163
WAP:Pten$^{f/f}$	0.0197
MMTV:Pten$^{f/f}$	0.0172
MMTV:p53$^{f/f}$	0.0121

TUNEL

Type	Pten$^{f/f}$:p53$^{f/f}$
Neu	0.994
Wnt1	0.724
WAP:Pten$^{f/f}$	0.043
MMTV:Pten$^{f/f}$	0.122
MMTV:p53$^{f/f}$	0.0344

Figure S5C-F

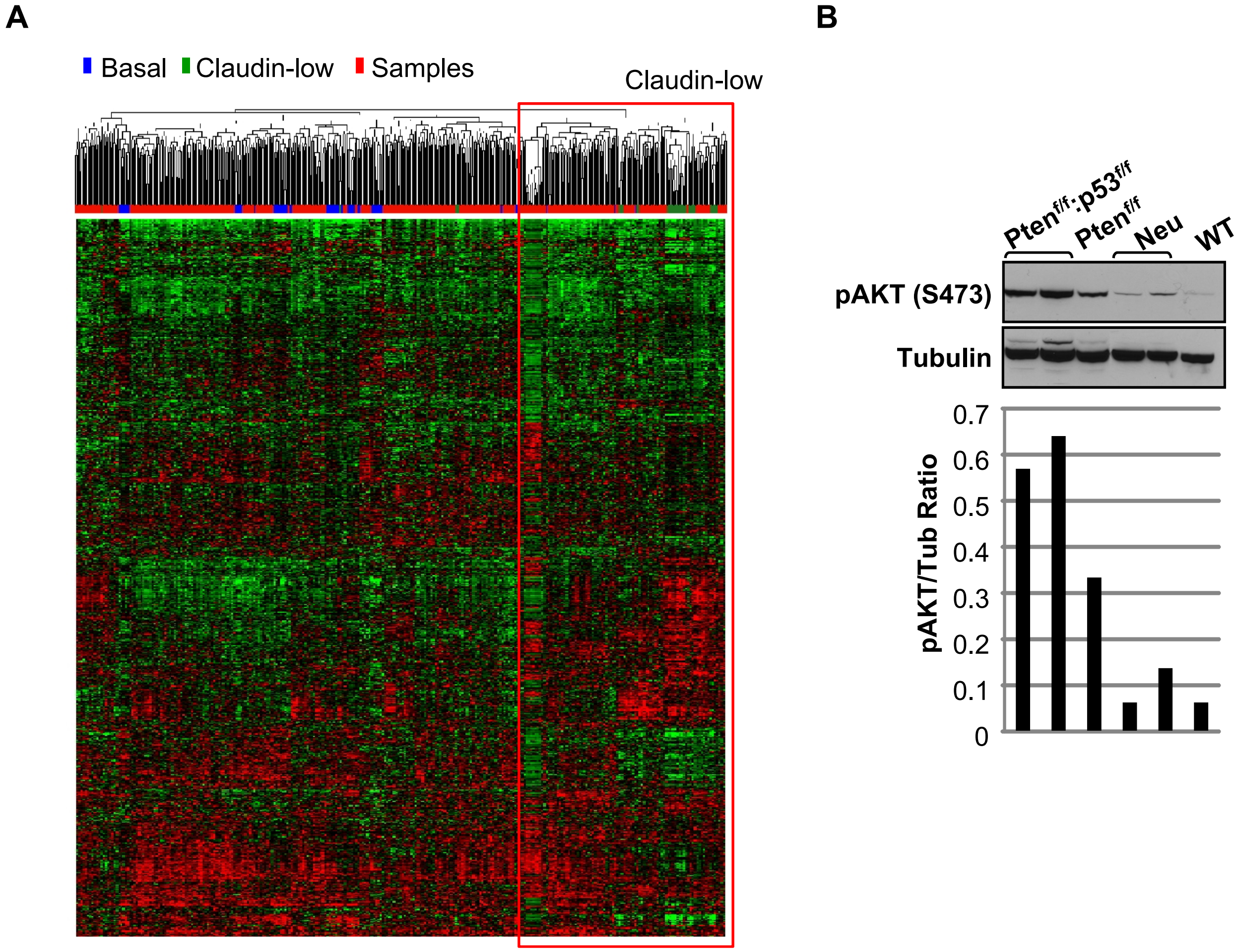

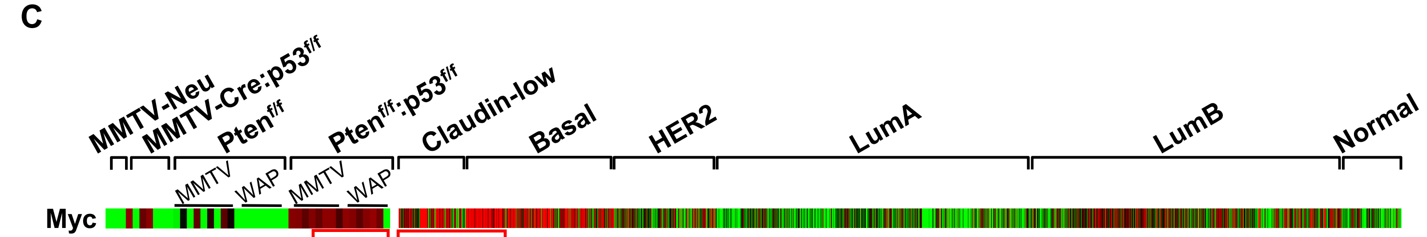

	Neu	p53	Pten	Pten: p53	Claudin-low	Basal	HER2	LumA	LumB	Normal
Ave.	0.18	0.56	0.40	0.85	0.63	0.70	0.48	0.37	0.51	0.35
p-value	0.0002	0.123	0.0065	--	--	0.00243	$2.03x10^{-5}$	$2.03x10^{-5}$	$2.04x10^{-5}$	$2.03x10^{-5}$

Figure S6

A

$Pten^{\Delta f}$:$p53^{\Delta f}$ tumor lines (4X)

Compound Name	Fold	T-Score
ER27319 maleate	-6.83	-19.96
FAK Inhibitor 14	-4.31	-8.85
Ryuvidine	-3.29	-8.17
**NH125**	**-3.14**	**-8.26**
NSC 663284	-2.94	-3.44
BIO	-2.82	-5.84
**TX-1918**	**-2.65**	**-2.20**
**BI 78D3**	**-2.62**	**-3.18**
K-252a, K-2151	-2.55	-2.54
Staurosporine	-2.42	-2.01
SB-505124	-2.34	-3.24
PKC-412	-2.26	-2.08
TAE-684	-2.22	-1.58
PKR inhibitor C16	-2.21	-1.66
PHA 665752	-2.21	-1.47
IKK 16	-2.15	-1.29
Lestaurtinib,	-2.11	-2.60
Chelerythrine chloride	-2.03	-1.10
**Other PI3K Pathway Specific Drugs**		
**A-443654**	**-1.94**	**-1.17**
**PIK-75**	**-1.92**	**-1.00**
**OSU-03012**	**-1.92**	**-1.35**
**PI-103**	**-1.74**	**-1.06**
**NVP-BEZ235**	**-1.70**	**-0.93**
**BIBW-2992**	**-1.66**	**-1.10**
**GDC-0941**	**-1.57**	**-0.89**
**API-2**	**-1.55**	**-1.23**
**NVP-AEW541**	**-1.54**	**-0.59**
**EKI-785**	**-1.51**	**-0.75**

BT549 & HCC1937

Compound Name	Fold	T-Score
FAK Inhibitor 14	-3.29	-394.74
AV-412	-3.23	-2795.85
**BI 78D3**	**-3.04**	**-87.48**
**TX-1918**	**-2.93**	**-1255.62**
Staurosporine	-2.75	-23.96
TCS 2312	-2.51	-35.37
MK-1775	-2.42	-36.32
**A-443654**	**-2.28**	**-3.85**
NSC 663284	-2.27	-9.57
**PIK-75**	**-2.26**	**-6.26**
Chelerythrine chloride	-2.23	-9.61
SNS-032	-2.17	-14.07
AZD-7762	-2.15	-4.76
**NH125**	**-2.12**	**-64.07**
Alvocidib	-2.11	-5.33
BMS-3	-2.10	-22.85
IKK 16	-2.08	-22.30
Alsterpaullone	-2.06	-20.55
**Other PI3K Pathway Specific Drugs**		
**NVP-AEW541**	**-1.69**	**-103.34**
**BIBW-2992**	**-1.58**	**-5.33**
**GDC-0941**	**-1.56**	**-18.44**
**NVP-BEZ235**	**-1.52**	**-1.6**
**EKI-785**	**-1.47**	**-28.48**
**PI-103**	**-1.42**	**-1.39**
**OSU-03012**	**-1.31**	**-1.03**
**API-2**	**-1.15**	**-0.9**

B

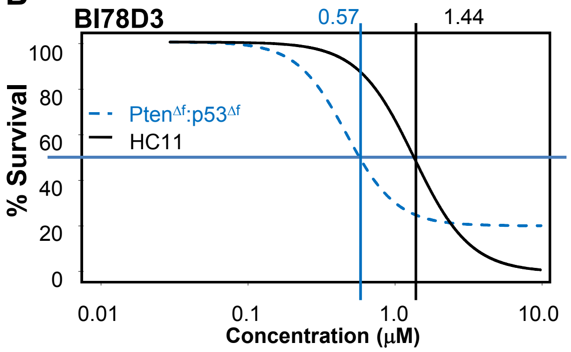

C

Path	TX-1918 r	TX-1918 p-value	Doxorubicin r	Doxorubicin p-value
**Akt**	**-0.7**	**0.0001**	**0.42**	**0.0128**
p53	-0.57	0.0001	0.54	0.0001
Src	-0.51	0.0001	-0.31	0.0849
E2F	-0.46	0.0006	0.19	0.332
TGFβ	-0.45	0.0008	-0.35	0.0414
PI3K	-0.35	0.0286	0.3	0.104
HER2	-0.35	0.0216	-0.04	0.83
βcat	-0.32	0.0381	0.55	0.0001
p63	-0.28	0.0591	0.52	0.0006
Ras	-0.16	0.304	-0.32	0.256
INFα	-0.14	0.382	0.44	0.0085
INFγ	-0.03	0.837	0.4	0.0184
PR	0.01	0.962	0.22	0.256
Stat3	0.63	0.0001	-0.54	0.0002
Myc	0.65	0.0001	-0.4	0.0204
EGFR	0.68	0.0001	-0.71	0.0001
ER	0.76	0.0001	-0.41	0.0172
TNFα	0.92	0.0001	-0.59	0.0001

Figure S7
